# New model of superior semicircular canal dehiscence with reversible diagnostic findings characteristic of patients with the disorder

**DOI:** 10.3389/fneur.2022.1035478

**Published:** 2023-01-19

**Authors:** P. Ashley Wackym, Carey D. Balaban, Olivia J. Van Osch, Brian T. Morris, Mark-Avery Tamakloe, Victoria L. Salvatore, Sudan Duwadi, Jennifer D. Gay, Todd M. Mowery

**Affiliations:** ^1^Department of Otolaryngology – Head and Neck Surgery, Rutgers Robert Wood Johnson Medical School, New Brunswick, NJ, United States; ^2^Rutgers Brain Health Institute, New Brunswick, NJ, United States; ^3^Departments of Otolaryngology, Neurobiology, Communication Sciences and Disorders, Bioengineering and Mechanical Engineering and Materials Science, University of Pittsburgh School of Medicine, Pittsburgh, PA, United States

**Keywords:** cognitive dysfunction, dizziness, memory, perilymph fistula, spatial disorientation, superior semicircular canal dehiscence, third window syndrome, vestibular evoked myogenic potential

## Abstract

**Background:**

Third window syndrome is a vestibular-cochlear disorder in humans in which a third mobile window of the otic capsule creates changes to the flow of sound pressure energy through the perilymph/endolymph. The nature and location of this third mobile window can occur at many different sites (or multiple sites); however, the most common third mobile window is superior semicircular canal dehiscence (SSCD). There are two essential objective diagnostic characteristics needed to validate a model of SSCD: the creation of a pseudoconductive hearing loss and cVEMP increased amplitude and decreased threshold.

**Methods:**

Adult Mongolian gerbils (*n* = 36) received surgical fenestration of the superior semicircular canal of the left inner ear. ABR and c+VEMP testing were carried out prior to surgery and over acute (small 1 mm SSCD, 1–10 days) or prolonged (large 2 mm SSCD, 28 days) recovery. Because recovery of function occurred quickly, condenser brightfield stereomicroscopic examination of the dehiscence site was carried out for the small SSCD animals *post-hoc* and compared to both ABRs and c+VEMPs. Micro-CT analysis was also completed with representative samples of control, day 3 and 10 post-SSCD animals.

**Results:**

The SSCD created a significant worsening of hearing thresholds of the left ear; especially in the lower frequency domain (1–4 kHz). Left (EXP)/right (CTL) ear comparisons *via* ABR show significant worsening thresholds at the same frequency representations, which is a proxy for the human pseudoconductive hearing loss seen in SSCD. For the c+VEMP measurements, increased amplitude of the sound-induced response (N1 2.5 ms and P1 3.2 ms) was observed in animals that received larger fenestrations. As the bone regrew, the c+VEMP and ABR responses returned toward preoperative values. For small SSCD animals, micro-CT data show that progressive osteoneogenesis results in resurfacing of the SSCD without bony obliteration.

**Conclusion:**

The large (2 mm) SSCD used in our gerbil model results in similar electrophysiologic findings observed in patients with SSCD. The changes observed also reverse and return to baseline as the SSCD heals by bone resurfacing (with the lumen intact). Hence, this model does not require a second surgical procedure to plug the SSCD.

## Introduction

While it was nearly a century ago that Tullio described the physiologic outcomes of creating a third mobile window in the semicircular canals of pigeons (1, 2), it was a quarter century ago that Minor et al. first described superior semicircular canal dehiscence (SSCD) in two patients (3). However, this is not a new clinical entity as SSCD has been observed after CT imaging of Egyptian mummy heads (4). Since 1998, many locations of third mobile windows have been described [for review see (5–7)]. The sound-induced dizziness and/or nystagmus due to a third mobile window has been memorialized by the eponym “Tullio phenomenon.” Clinically, the most thoroughly characterized third mobile window is SSCD. Minor later reported a conductive hearing loss, which was recognized as a pseudoconductive hearing loss (bone-conduction hyperacusis) (8), as well as a reduced cervical vestibular myogenic potential (cVEMP) threshold in patients with SSCD to 81 ± 9 dB nHL (9). We, and others, have proposed the more general term of third window syndrome because the same spectrum of symptoms, signs on physical examination, and audiological diagnostic findings, e.g., pseudoconductive hearing loss and the abnormally reduced cVEMP threshold with increased amplitude, are encountered with SSCD as well as the other 14 known third mobile window locations that can be seen with high-resolution temporal bone CT [for review see (5–7)].

There are two essential objective diagnostic characteristics needed to validate a model of SSCD: the creation of a pseudoconductive hearing loss and VEMP increased amplitude and decreased threshold. Because unilateral bone-conduction ABR thresholds in rodents cannot be measured, it is not possible to measure the pseudoconductive hearing loss, but the proxy finding of worsened ABR thresholds is adequate, particularly, when the hearing loss recovers once the surgically created SSCD recloses after bone regrowth. The two previous animal models tried to record cVEMP responses, but they did not appreciate that the sternocleidomastoid evoked potentials (cVEMP) are relaxation potentials, and this explains why the animal SSCD literature has no convincing cVEMP responses shown in the models published to date (10–12). In contrast, it is well-known that sound-induced otolithic neck extensor responses are excitatory potentials (13); therefore, we developed a c+VEMP method that demonstrates increased c+VEMP amplitudes and decreased c+VEMP thresholds after surgically creating a SSCD in our model, just as we see in patients with SSCD.

Patients with third window syndrome, including SSCD, also report symptoms of autophony, cognitive dysfunction, spatial disorientation, anxiety, and autonomic dysfunction (7, 14–16). To varying degrees, patients with SSCD describe cognitive dysfunction (impaired memory and concentration, word finding and name finding difficulty, occasional slurred speech and for women, the loss of the ability to listen to more than one person speaking at a time), spatial disorientation (trouble judging distances, sense of detachment, sometimes perceiving the walls moving/breathing or the floor moving, and less commonly out of body experiences), and anxiety (sense of impending doom). In children and young adults continuing their education, their academic performance typically drops; they miss days of school and are often assigned a psychiatric or neurobehavioral diagnosis (5–7, 14–16). SSCD and other third window syndrome patients with other sites of dehiscence frequently experience migraine headaches as well as the three variants of migraine: ocular migraine [an older term which has been replaced with migraine with aura (bilateral visual field loss) with or without headache], hemiplegic migraine, and vestibular migraine (5–7, 14–19). Retinal migraine (unilateral visual field loss) has not been reported in patients with SSCD or other third window syndrome sites of dehiscence. Ward et al. reported that many patients with SSCD also have migraine, but they commented that this may represent the high prevalence of migraine in the general population and that SSCD is an effective migraine trigger (19). Naert et al. completed a systematic review of SSCD symptoms and after combining synonymous terms, 22 symptoms were derived by consensus that also included headache (20). These additional symptoms typically resolve or markedly improve after surgically managing the third mobile window (5–7, 14–19, 21, 22). However, for reasons that are not understood, the pseudoconductive hearing loss typically does not resolve after plugging the SSCD (23–25). The value of developing an animal model of SSCD in a rodent well-known for inner ear similarities with humans and capability to be trained to perform complex behavioral tasks designed to measure decision-making is that the basic electrophysiologic, neural plasticity, and molecular events subserving the cognitive dysfunction can be studied and understood. With this knowledge, new translational interventions to treat patients with SSCD can be discovered. It was for this purpose that we sought to develop this SSCD model in adult Mongolian gerbils. Consequently, we hypothesized that the creation of bony dehiscence of the SSC would result in diagnostic findings that resemble patients with SSCD. For example, we expect to see changes in otolithic stimulation as measured by VEMPs and pseudoconductive hearing loss as measured by ABR as a proxy since bone-conduction thresholds cannot be measured in gerbils due to small skull size.

## Materials and methods

### Animals

A total of 36 adult Mongolian gerbils *Meriones unguiculatus* (19 males and 17 females) were used in this study. All animals were housed in the same vivarium facility under a 12/12 dark cycle with *ad libitum* access to food and water. Animals were randomly divided into two groups, which received either small (1 mm) or large (2 mm) semicircular canal fenestrations. The Rutgers University IACUC reviewed and approved this research protocol (PROTO202000179).

### Surgical creation of the superior semicircular canal dehiscence

**Figure 1:**
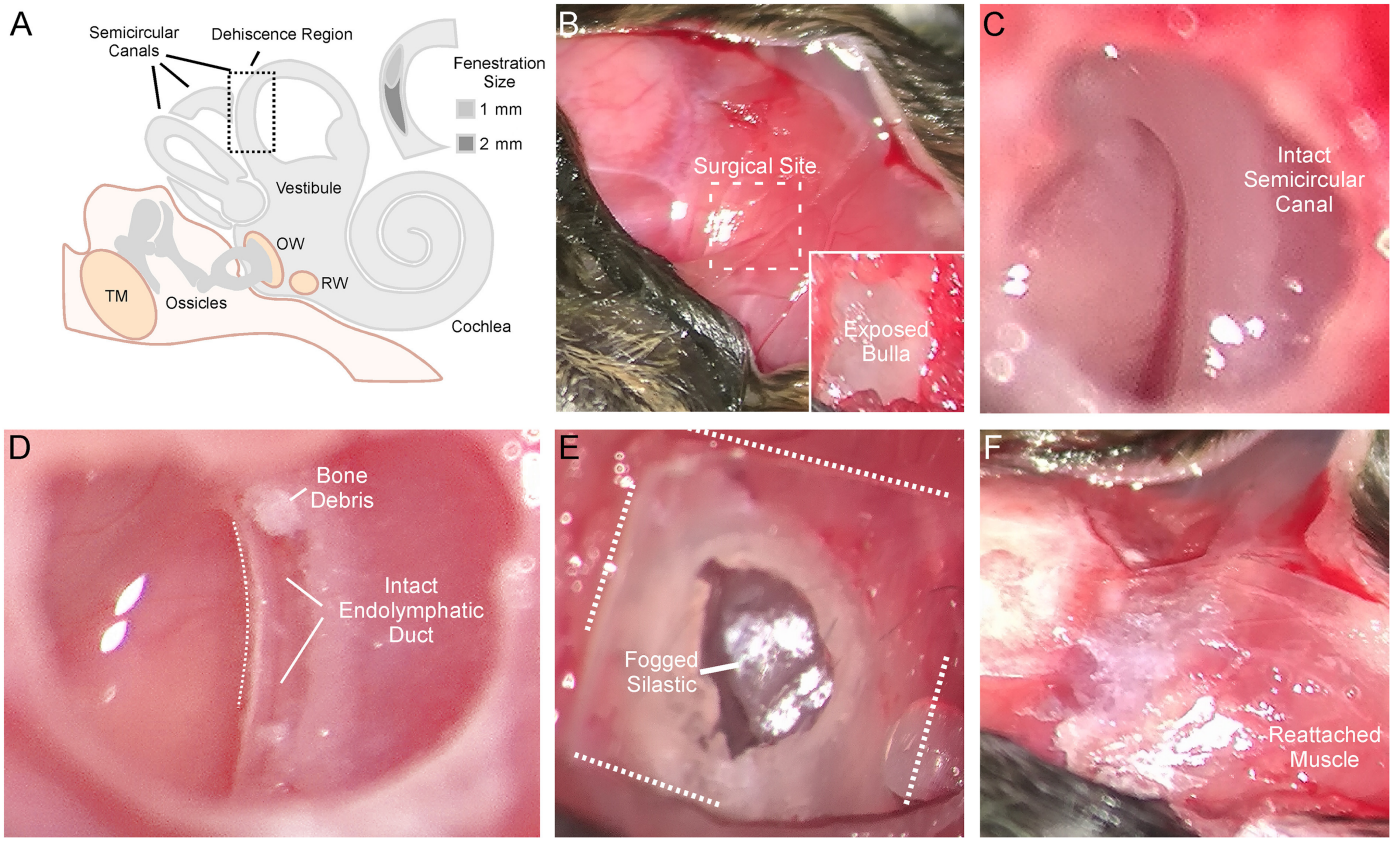
Detailed surgical method for creating left semicircular canal dehiscence (SSCD). **(A)** Cartoon showing the middle/inner ear and the location of the surgically created SSCD “third mobile window.” The inset shows the dehiscence site. **(B)** Photomicrograph showing the exposed skull (top left). The exposed nuchal muscles can be seen (center and bottom right). In the inset, after the muscles are taken down from the bulla, the superior bulla is exposed. **(C)** The superior bulla is opened widely with a 1.5-mm diamond bur and the dissection is carried near, but not into the surrounding sinus, to avoid bleeding. Through the open bulla, the intact superior semicircular canal can be seen. **(D)** Using a 1-mm diamond bur, a controlled fenestration of the superior (anterior) semicircular canal is completed. It should be noted that the bone of the superior semicircular canal, including the endosteum, is precisely removed. The endolymphatic duct can be seen to be intact illustrating the precise surgical creation of the SSCD. Sterile physiologic saline is used to irrigate away bone debris. Note, bone debris at the margins of the fenestration cannot be removed by irrigation. This no doubt contributes to bone regrowth. The perilymph is open/exposed just as patients with SSCD have their perilymph exposed. **(E)** Photomicrograph shows the sealing of the bulla with Silastic to partition the air-filled bulla from the overlying neck muscles thereby restoring the normally air-filled middle ear. Note condensation on the interior surface of the Silastic seal. **(F)** The reattached muscles are glued to the skull with Medbond. This allows c+VEMP testing beginning the day after the surgical creation of the SSCD.

Animals were anesthetized with isoflurane and prepared for stereotaxic surgery. Figure 1 shows the detailed surgical method for creating a left SSCD. Figure 1A is a cartoon that shows the middle/inner ear and the location of the surgically created SSCD “third mobile window.” The inset (Figure 1A) shows the site of dehiscence. The skull (top left) and nuchal muscles (center and bottom right) are exposed (Figure 1B). After the muscles were taken down from the bulla, the superior bulla is exposed (Figure 1B, inset). The superior bulla was opened widely with a 1.5-mm diamond bur, and the dissection was carried near, but not into the surrounding sinus, to avoid bleeding (Figure 1C). Through the open bulla, the intact superior semicircular canal was visualized. Using a 1-mm diamond bur, a controlled fenestration of the superior (anterior) semicircular canal was completed (Figure 1D). It should be noted that the bone of the superior semicircular canal, including the endosteum, was precisely removed. The endolymphatic duct was seen to be intact and surrounded by perilymph illustrating the precise surgical creation of the SSCD (Figure 1D). Sterile physiologic saline was used to irrigate away bone debris; however, it was not possible to remove the bone debris at the margins of the fenestration by irrigation, likely contributing to the bone regrowth. After the fenestration was completed, the perilymph was open and exposed without egressing, just as patients with SSCD have their perilymph exposed. The open bulla was then sealed/partitioned with Sterile Silastic (Dow Chemical Company, Midland, MI) to partition the air-filled bulla from the overlying neck muscles thereby restoring the normally air-filled middle ear (Figure 1E) and avoiding a true conductive hearing loss. Condensation on the interior surface of the Silastic seal was deemed indicative of this restoration of function. Finally, the reattached muscles were glued to the skull with Medbond tissue glue (Stoelting Co., Wood Dale, IL), which allowed c+VEMP testing as soon as the day after the surgical creation of the SSCD (Figure 1F). Condenser brightfield stereomicroscope and micro-CT analysis were performed *post-hoc* to assess the SSCD site and bone regrowth.

### Auditory brainstem responses

**Figure 2:**
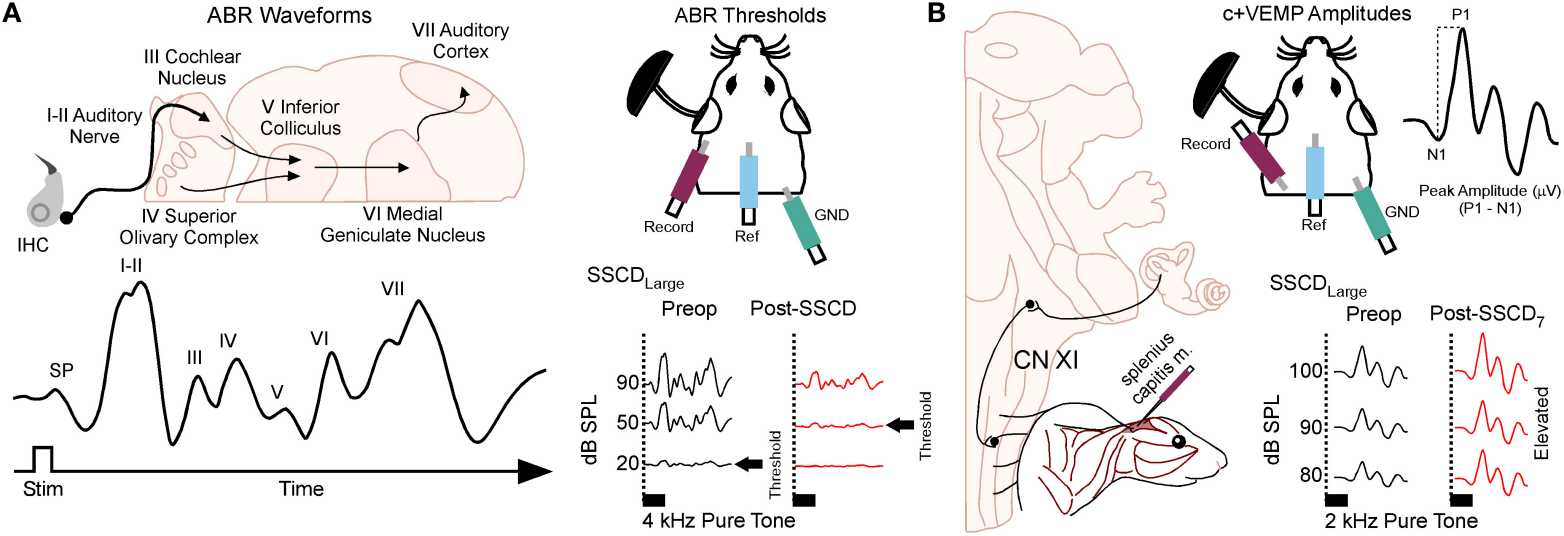
Semicircular canal dehiscence induces sound-induced changes to auditory and vestibular physiology. **(A)** Cartoon showing the configuration for closed field auditory brainstem responses (ABR) and threshold shifts that occur for 2 kHz tones after SSCD of the left ear. **(B)** Cartoon showing the configuration for the left ear when measuring the sound-induced cervical positive vestibular evoked myogenic potential (c+VEMP) in the anesthetized animal. Here, electrodes are inserted into the splenius capitus extensor muscles, and sound is delivered in a closed field to the ear with SSCD. The c+VEMP amplitude is calculated by subtracting the positive P wave 1 from the negative N wave 1. Pictured is the increase in c+VEMP amplitudes 7 days after surgical creation of a large SSCD.

Animals were anesthetized with isoflurane (1.0%) and placed in a small sound chamber (IAC, Sound Room Solutions, Inc, Glen Cove, NY). As shown in Figure 2A, ABR recordings were made by inserting pin electrodes subcutaneously at the vertex of the skull and just caudal to the right pinna; the ground electrode was inserted into the base of the tail. BioSigRZ software and the TDT ABR system (Tucker-Davis Technologies, Alachua, FL) were used to collect ABR data. A 10-cm tube (closed field) was inserted into the ear and placed at the opening of the ear canal. The left ear of the animal was stimulated *via* multi-field speaker (MF1, Tucker-Davis Technologies, Alachua, FL) at 1, 2, 4, 8, and 16 kHz tones [90 to 20 dB SPL (10 dB steps)], 5 ms, 2 ms linear ramp rise-fall times at 25 Hz. Traces were averaged across 500 (threshold) sweeps. Thresholds for each frequency were measured as the last dB SPL (i.e., 10 dB SPL resolution stimulus level) that elicited a tone-induced ABR.

### Sound-induced cervical positive potential vestibular evoked myogenic potentials

As shown in Figure 2B, sound-induced otolithic stimulation and evoked intramuscular excitatory potential recordings were made by inserting pin electrodes into the neck extensor muscles (splenius capitus m.) and the reference electrode in the vertex of the skull. BioSigRZ software and the TDT ABR system were used to collect c+VEMP data. A 10-cm tube capable of delivering 100 dB SPL (see TDT specs, Closed Field) was inserted into the ear and placed at the opening of the ear canal. The left ear of the animal was stimulated *via* multi-field speaker (MF1, Tucker-Davis Technologies, Alachua, FL) at 2kHz [100 to 80 dB SPL (5 dB steps), 5 ms, 2 ms linear ramp rise-fall times sampled at 25 kHz]. Traces were averaged across 500 (threshold) sweeps. The c+VEMPs were recorded under low-isoflurane anesthesia (<1.5%), near conditions of wakefulness. The c+VEMP was measured when it appeared under the condition of stimulation of air-conducted sound at 2 kHz and 100 dB. Peak amplitudes were measured by subtracting the peak of the negative N1 wave (in μV) from the later positive P1 wave (see Supplementary Figure 1).

### Condenser brightfield stereomicroscope

After the systematic ABR and c+VEMP recordings were collected, animals were euthanized (Euthasol 300 mg/kg) and perfused for histology. Each animal's heart was accessed through the diaphragm. The right atrium was cut, and 20 ml of room-temperature phosphate-buffered saline (1M) was perfused through the left ventricle. This was followed by 20 ml of cold paraformaldehyde (4%). After perfusion, the animals were decapitated. The left bulla was dissected and immersed in paraformaldehyde (4%). The superior (anterior) semicircular canal was imaged using a condenser brightfield stereomicroscope through the opening into the bulla on a Revolve R4 microscope (ECHO, San Diego, CA).

### Micro-CT imaging

The bullae containing the inner ears of three adult gerbils (>P86) were dissected, fixed with paraformaldehyde (4%), and then stored in phosphate-buffered saline (1.0M). Serial micro-CT images of the specimens were acquired in the sagittal plane at a slice thickness of 5.0 μm [Bruker Skyscan 1,172 nanoCT (Bruker Corporation, Allentown, PA, USA)]. The high-resolution allows delineating of the semicircular canals and other inner ear structures. The 3D reconstructed images were rotated and oriented into the natural anatomical position. Micro-CT with maximum intensity projections (MIP), micro-CT with attenuated images, and serial section review through all six planes in the rectangular grid were created and reviewed using the Brucker CTvox software (Bruker Corporation). Specific regions of interest were selected, imaged, and exported for Figure creation in Canvas X (Canvas GFX, Inc, Boston, MA, USA).

### Statistical analyses

Statistical analyses were performed using JMP software (SAS, Carey, NC) and SPSS (IBM, Armonk, New York). Figures were generated using JMP software. To test the main effects of SSCD over postoperative days, mixed model ANOVAs were generated. Where appropriate the Greenhouse–Geisser Adjustment test, the Kolmogorov–Smirnov tests, the Lilliefors Corrections, the Bonferroni corrections, and the Least Significant differences (LSD) multiple range tests were used to explore frequency, amplitude, and day-by-day interaction effects associated with the main effects. When comparing two unmatched data sets within groups (e.g., baseline to postoperative day), Student's *t*-test was utilized, and where appropriate *post-hoc* analysis using Tukey's Honestly Significant Difference (HSD) test was used to account for variance within and between groups. A matched *t*-test modeling was used for matched pair data sets that compared preoperative data to endpoint data within groups. A linear regression analysis was used to calculate adjusted *R*^2^ scores when correlating data within groups. The mixed model ANOVA with repeated measures was used to analyze data between groups over days. For all analyses, significance was determined at *p* < 0.05 or greater. Data in the figures display group mean ± SEM or actual data points (e.g., regression analysis).

## Results

### Establishment of superior semicircular canal dehiscence induced changes to cochlear and vestibular output

We measured ABRs (Figure 2A) in animals (*n* = 10, 5M, 5F) with surgically created small (1 mm) SSCD fenestrations. In our pilot experiments, we found significant increases in ABR threshold compared to preoperative baselines 3 days after SSCD for all animals (mean ± SEM; baseline: 43.0 ± 8.8 vs. post-SSCD day 3: 67.25 ± 15.8; *t* = 1.99, *p* < 0.0001, Supplementary Figure 1A) suggesting that a pseudoconductive hearing loss accompanies SSCD in our model as it does in humans.

For the c+VEMP studies (Figure 2B), we first established the latency of N and P waves at descending sound intensities, as each organism differs in latency of nerve conduction based on size (e.g., human p latency is 13 ms and n latency is 23 ms, see Supplementary Figiure 1B). As we observed from ABR measurements, c+VEMP characteristics [N1 latency (2.5 ms average), P1 latency (3.2 ms average), Supplementary Figure 1C; wave 1 duration, Supplementary Figure 1D] also were significantly altered by the SSCD.

#### Preoperative ABR and c+VEMPs

The preoperative ABR thresholds [*F*_(1,8)_ = 3.27, NS] and c+VEMPs [*F*_(1,8)_ = 0.00, NS] did not differ significantly between the animals that received small or large SSCD. The preoperative measurements of ABR thresholds showed a pattern of significant threshold differences as a function of frequency [F_(df = 2.19, 17.52)_ = 26.07, *p* < 0.001, Greenhouse–Geisser adjustment], The minimum threshold of 20 dB SPL was recorded from all subjects at 4 kHz: it was significantly lower (*p* < 0.05, Bonferroni correction) than the thresholds of 32.0 ± 2.0 dB SPL at 1 kHz, 32.0 ± 2.1 dB SPL at 8 kHz, and 43.0 ± 2.0 dB SPL at 16 kHz (mean ± SE). There were no significant variations in c+VEMP amplitudes across intensities in the preoperative assessments. The preoperative c+VEMP values at 90 dB SPL and 100 dB SPL stimuli were consistent with single Gaussian distributions (Kolmogorov–Smirnov tests and Lilliefors correction) with mean amplitudes and standard deviations of 604.72 ± 112.78 μV and 706.11 ± 159.71 μV, respectively.

### Tracking prolonged peripheral impairment, recovery, and persistence after large superior semicircular canal dehiscence

The small SSCD group data showed a significant impairment in ABR and c+VEMP physiology. The effects were consistent with the major variability between animals being the latency of return toward baseline within 1 week of surgery (Table 1) [see supplementary results (Supplementary Figures 2, 3)]. Therefore, we hypothesized that larger fenestrations could expand this timeline and more appropriately map onto the human disorder. For this experiment, five animals (3M, 2F) had microsurgical creation of large SSCDs (2 mm) followed by ABR and c+VEMP measurements throughout 28 days of recovery. Recordings were carried out prior to surgery and 7, 14, 21, and 28 days after the microsurgical creation of the large SSCD. An analysis was carried out within animals across days and between animals across days.

**Table 1:** Recovery of ABR and c+VEMPs after Small SSCD.

**1A: Small SSCD, ABR**.					
	**Post-SSCD 1**	**Post-SSCD 3**	**Post-SSCD 5**	**Post-SSCD 7**	**Post-SSCD 10**
Subject 1	*p* < 0.0001	*p* < 0.0001	*p* = 0.0006	*p* = 0.004	*p* = 0.099
Subject 2	*p* = 0.032	*p* = 0.016	*p* = 0.036	*p* = 0.14	*p* = 0.070
Subject 3	*p* = 0.004	*p* = 0.0003	*p* = 0.0005	*p* = 0.0010	*p* = 0.019
Subject 4	*p* = 0.010	*p* = 0.0006	*p* = 0.0028	*p* = 0.034	*p* = 0.099
Subject 5	*p* < 0.0001	*p* = 0.0041	*p* = 0.016	*p* = 0.117	*p* = 0.37
**1B: Small SSCD, c**+**VEMP**.					
	**Post-SSCD 1**	**Post-SSCD 3**	**Post-SSCD 5**	**Post-SSCD 7**	**Post-SSCD 10**
Subject 1	*p* = 0.0005	*p* = 0.0005	*p* = 0.053	*p* = 0.93	*p* = 0.23
Subject 2	*p* = 0.0011	*p* = 0.086	*p* = 0.28	*p* = 0.095	*p* = 0.102
Subject 3	*p* = 0.00015	*p* = 0.00011	*p* = 0.0003	*p* = 0.0002	*p* = 0.0234
Subject 4	*p* < 0.0001	*p* < 0.0001	*p* = 0.0007	*p* = 0.008	*p* = 0.0013
Subject 5	*p* = 0.0027	*p* = 0.0002	*p* = 0.65	*p* = 0.70	*p* = 0.88

ABR, auditory brainstem response; c+VEMP, cervical positive vestibular evoked myogenic potentials; SSCD, superior semicircular canal dehiscence.

**Figure 3:**
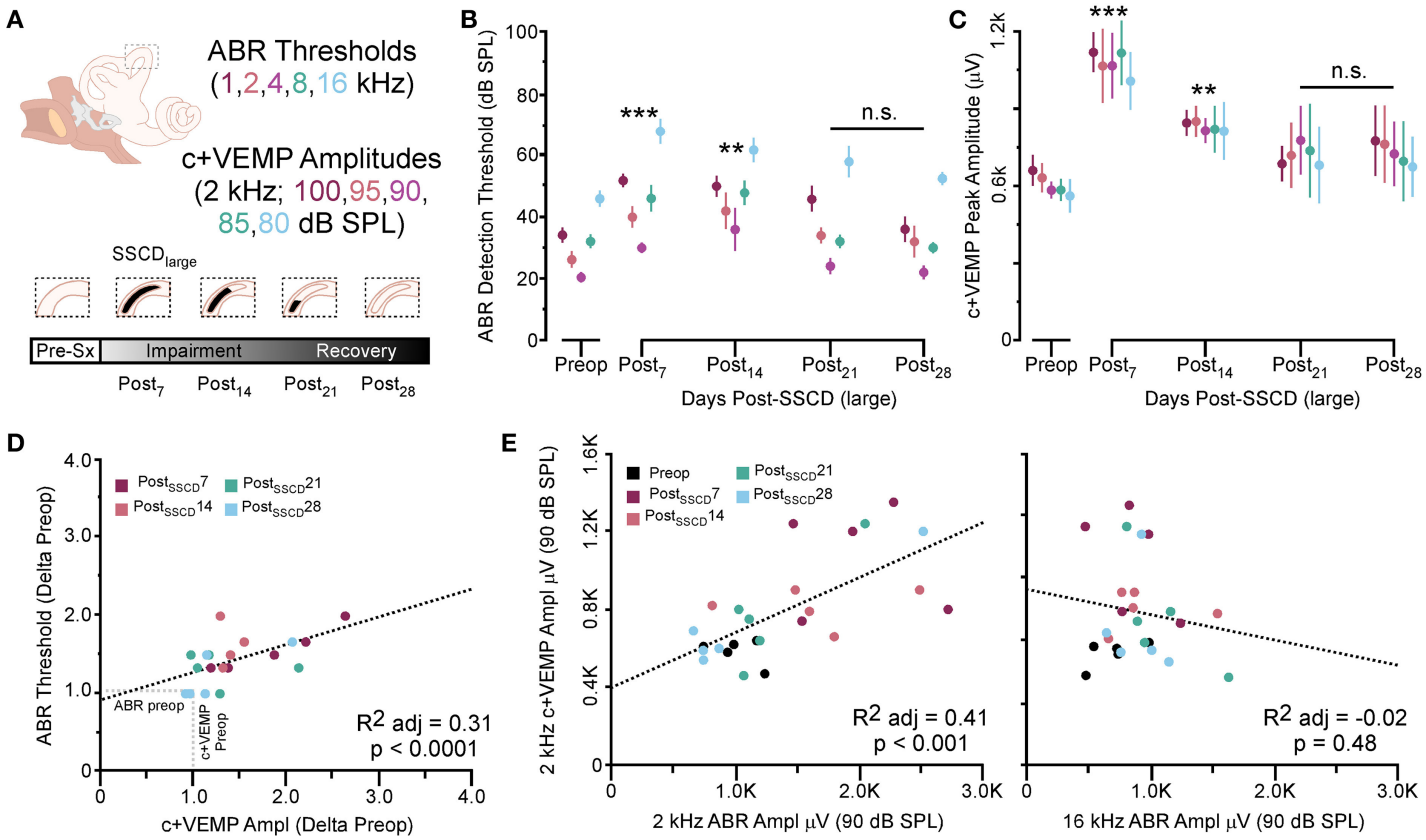
The correlated group changes to sound-induced ABR thresholds and c+VEMP amplitudes after large SSCD. **(A)** Diagram showing the theoretical recovery of ABR thresholds and c+VEMP amplitudes over days (post-SSCD 7, 14, 21, and 28). **(B)** Line chart shows the group means for ABR thresholds over postoperative days in animals that received the large SSCD. **(C)** Line chart shows the group means for c+VEMP amplitudes over post-SSCD days in animals that received the large SSCD. **(D)** Scatterplot showing a significant negative correlation between auditory thresholds and c+VEMP amplitudes throughout impairment and recovery from large SSCD. **(E)** Scatterplot showing a significant positive correlation between c+VEMP amplitudes and ABR amplitudes at 2 kHz (90 dB SPL) and a non-significant correlation between c+VEMP amplitudes and ABR amplitudes at 16 kHz (90 dB SPL) for large SSCD. **p ≤ 0.001, ***p ≤ 0.0001. ns, not significant.

As with the smaller fenestrations, there was significant variability between individual animals in the duration of the air-conduction increased ABR threshold as a proxy for the pseudoconductive hearing loss seen in patients with SSCD (Supplementary Figure 4). There was a general trend across frequencies in individual subjects such that ABR thresholds increased over the first 2 weeks followed by a return toward baseline over the next 2 weeks (Supplementary Figures 4B–F). The grand mean (Figure 3) of thresholds (across frequencies) was elevated significantly from the preoperative baseline on post-SSCD day 14 (*p* = 0.05) and reduced significantly from preoperative levels on post-SSCD day 28 (*p* < 0.01). LSD multiple range tests were used to make direct comparisons between pre-SSCD thresholds at each frequency (baseline) and the various day-by-day threshold changes post-SSCD. The time course of threshold changes varied with frequency. At 1 kHz, there were significant threshold decreases from the preoperative assessment at 7 days (*p* < 0.01), 14 days (*p* < 0.01), and 21 days (*p* < 0.01) after the creation of the large SCCD. The same pattern appeared at 2 kHz, with significant decreases from the preoperative baseline threshold at post-SSCD 7 days (*p* < 0.01), 14 days (*p* < 0.05), and 21 days (*p* < 0.05). At 8 kHz, though, significant threshold decrements (relative to preoperative value) were only found at 7 days (*p* < 0.01) and 14 days (*p* < 0.05) after the surgery, followed by a recovery to baseline. The pattern was the same at 16 kHz; significant reductions from the preoperative baseline were identified at 7 days (*p* < 0.01) and 14 days (*p* < 0.05) after the creation of the large SCCD. Table 2A shows the means and SEMs and significance tests for ABR thresholds in each individual animal that received large SSCD fenestrations.

**Table 2:** Recovery of ABR and c+VEMPs after Large SSCD.

**2A: Large SSCD, ABR**.				
	**Post-SSCD 7**	**Post-SSCD 14**	**Post-SSCD 21**	**Post-SSCD 28**
Subject 1	*p* = 0.016	*p* = 0.019	*p* = 0.373	*p* = 0.495
Subject 2	*p* = 0.016	*p* = 0.003	*p* = 0.016	*p* = 0.032
Subject 3	*p* = 0.008	*p* = 0.024	*p* = 0.37	*p* = 0.37
Subject 4	*p* = 0.004	*p* = 0.004	*p* = 0.70	*p* = 0.37
Subject 5	*p* = 0.024	*p* = 0.034	*p* = 0.099	*p* = 0.37
**2B: Large SSCD, c**+**VEMP**.				
	**Post-SSCD 7**	**Post-SSCD 14**	**Post-SSCD 21**	**Post-SSCD 28**
Subject 1	*p* < 0.0001	*p* = 0.0015	*p* = 0.79	*p* = 0.55
Subject 2	*p* = 0.006	*p* = 0.002	*p* = 0.006	*p* = 0.0001
Subject 3	*p* = 0.014	*p* = 0.45	*p* = 0.77	*p* = 0.14
Subject 4	*p* = 0.004	*p* = 0.010	*p* = 0.11	*p* = 0.10
Subject 5	*p* = 0.003	*p* = 0.0018	*p* = 0.085	*p* = 0.062

ABR, auditory brainstem response; c+VEMP, cervical positive vestibular evoked myogenic potentials; SSCD, superior semicircular canal dehiscence.

**Figure 4:**
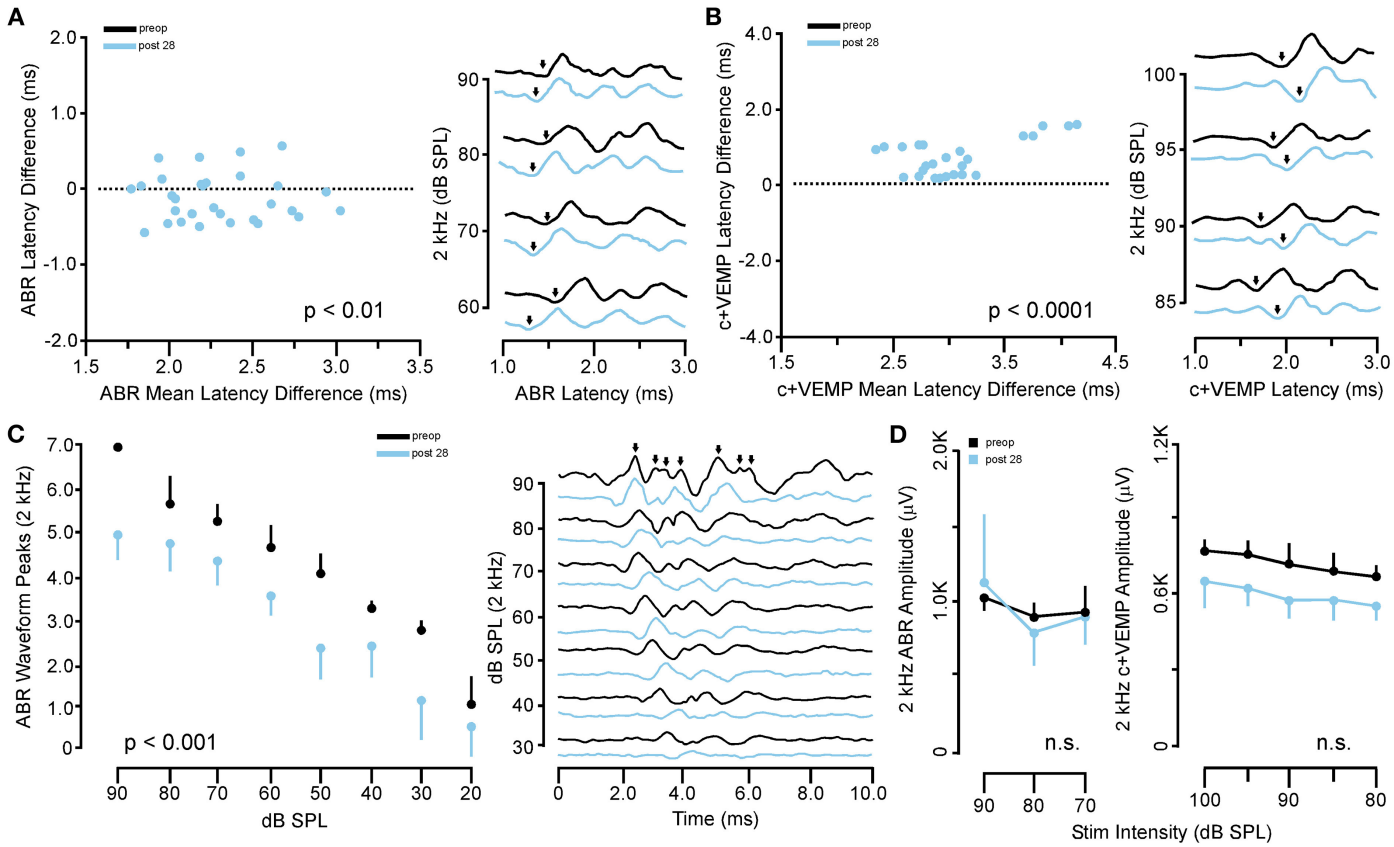
Persistent changes to ABR and c+VEMP waveform latency and expression after recovery from SSCD. **(A)** Scatterplot (left) showing the matched pair analysis of wave 1 ABR latency before SSCD (preoperative) and after recovery for the large SSCD group (post-SSCD 28) SSCD groups. A representative example of the persistent increase in ABR latency is shown on the right. **(B)** Scatterplot (left) showing the matched pair analysis of wave 1 c+VEMP latency before SSCD (preoperative) and after recovery for the large SSCD group (post-SSCD 28). A representative example of the persistent decrease in c+VEMP latency is shown on the right. **(C)** Line plot (left) comparing the average number of ABR waveform peaks present for 2 kHz stimuli at each dB SPL before (preoperative) and after recovery for large SSCD group (post-SSCD 28). Diagram (right) showing a representative example of ABR waveforms for 2 kHz stimuli before (preoperative) and after recovery (post-SSCD 28, dashed line) from large SSCD. **(D)** Left, line plot comparing ABR amplitudes between preoperative baselines and end point recordings for large SSCD group (post-SSCD 28) at 2 kHz stimulation levels. Right, line plot comparing c+VEMP amplitudes between preoperative baselines and end point recordings for large SSCD group (post-SSCD 28).

In the same animals, c+VEMPs were measured after ABRs on each post-SSCD day. As with ABRs, the duration of changes in the peak amplitude varied between gerbils (Supplementary Figures 4H–L). LSD multiple range tests were used to make direct comparisons between preoperative peak amplitudes at 100 to 80 dB SPL stimulation (baseline) and the various day-by-day changes to peak amplitude after SSCD (Table 2B). The average c+VEMP amplitudes across frequencies were elevated significantly from preoperative values on post-SSCD day 7 (*p* < 0.05) and day 14 (*p* < 0.05), followed by a return to baseline levels. Subject 2 showed a continued impairment at post-SSCD day 28. Interestingly, the larger SSCD produced a sound-induced response (increased amplitude) in the opposite direction from the small SSCD response but is more similar to the cVEMP response in humans with SSCD that typically show larger cVEMP amplitudes and reduced thresholds in the affected ear. Table 2B shows the means and SEMs and significance tests for c+VEMP amplitudes in each individual animal that received large SSCD fenestrations. Between animal comparisons (Figure 3C) with repeated measures, ANOVA and LSD multiple range tests showed a significant increase in peak amplitude of c+VEMPs after post-SSCD 7 days at 80, 85, 90, 95, and 100 dB SPL (*p* < 0.05), which persisted at 14 days for stimulation at 90, 85, and 100 dB SPL (*p* < 0.05). The values did not differ from baseline after either 21 days or 28 days. As in the small SSCD group, the relationship between the ABR amplitudes at 2 kHz and the c+VEMP amplitudes was explored with regression analysis. Results are shown in line charts for the 2 kHz data in Figure 3B and the 90 dB SPL c+VEMP data in Figure 3C. For this analysis, ratios were calculated to show changes above (>1.0) or below (<1.0) preoperative baselines. The relationship is linear [c+VEMP (μV) = 0.91 + 0.35^*^ABR Threshold (dB), adjusted *R*^2^ = 0.29, *p* < 0.01]. Again, this correlation suggested a physiological inner ear coupling between the effects on sensory (ABR) and motor (c+VEMP) consequences of the large SCCD. The correlation between c+VEMP amplitudes at 90 dB SPL (Figure 3C) and ABR VEMP amplitudes at 90 dB SPL for 2 kHz and 16 kHz frequency measurements are shown in Figure 3B. For the 2 kHz correlation (Figure 3E, left), there was a significant linear relationship [c+VEMP (μV) = 401 + 0.27^*^ABR Amplitude (μV), adjusted *R*^2^ = 0.41, *p* < 0.001]. Like the small SSCD, the 16 kHz correlation (Figure 3F, right) did not show a linear relationship between c+VEMP amplitude and ABR amplitude at 90 dB SPL [c+VEMP (μV) = 912–0.13^*^ABR Amplitude (μV), adjusted *R*^2^ = −0.02, *p* = 0.48]. The comparison of the ABR threshold, ABR amplitude and latency, and c+VEMP amplitude, as well as latency showed a bimodal distribution of data around day 7 (see Supplementary Figures 5A–D and corresponding results). A clear relationship between elevated ABR thresholds and amplitude and c+VEMP amplitude emerges when data are grouped above and below thresholds of 40 dB SPL at 2 kHz based on SSCD size (see Supplementary Figures 5E–G and corresponding results). Lower thresholds were correlated with baseline and return to baseline, while increased thresholds were associated with significant impairments. Inspection of the waveforms in threshold-recovered animals suggested that there could be perseverative changes to ABR and c+VEMP latency. Therefore, we wanted to verify that returning thresholds were accompanied by similar returns in other physiological parameters. End-point analysis of the ABR and c+VEMP latency data suggested that persistent changes can remain after ABR thresholds, and c+VEMP amplitudes have returned to preoperative-like levels (Figure 3). Figure 4 compares preoperative waveform analysis data to ABR and c+VEMP data collected on the day of terminal recording. In Figure 4A, a matched pairs *t*-test demonstrated a significant decrease in superior semicircular canal wave 1 ABR latency at 2 kHz (preoperative latency vs. recovered latency: mean difference ± SEM; 0.165 ± 0.054; *t* = 3.02, *p* < 0.01). The c+VEMP N1 latency was significantly increased (matched pairs *t*-test: mean difference ± SEM; 0.70 ± 0.094, *t* = 7.4, *p* < 0.0001) after threshold recovery (Figure 4B). Inspection of the ABR waveform peaks (Figure 4C) showed a significant decrease in the number of identifiable peaks after threshold recovery [MANOVA with repeated measures; *F*_(1,13)_ = 29.1, *p* < 0.001]. Figure 4C shows the regression analysis of preoperative and endpoint ABR amplitudes at 2 kHz stimulation, as well as c+VEMP amplitude for large SSCD animals. There is no significant difference for ABR amplitudes at 2 kHz in large SSCD animals [*F*_(1,8)_ = 0.008, *p* = 0.98] or for c+VEMP amplitudes [*F*_(1,8)_ = 0.776, *p* = 0.40]. Thus, the changes in ABR/c+VEMP amplitudes do resolve with the return of ABR thresholds.

### Condenser brightfield stereomicroscope: Evidence of bone regrowth correlates with returns to ABR threshold baseline

**Figure 5:**
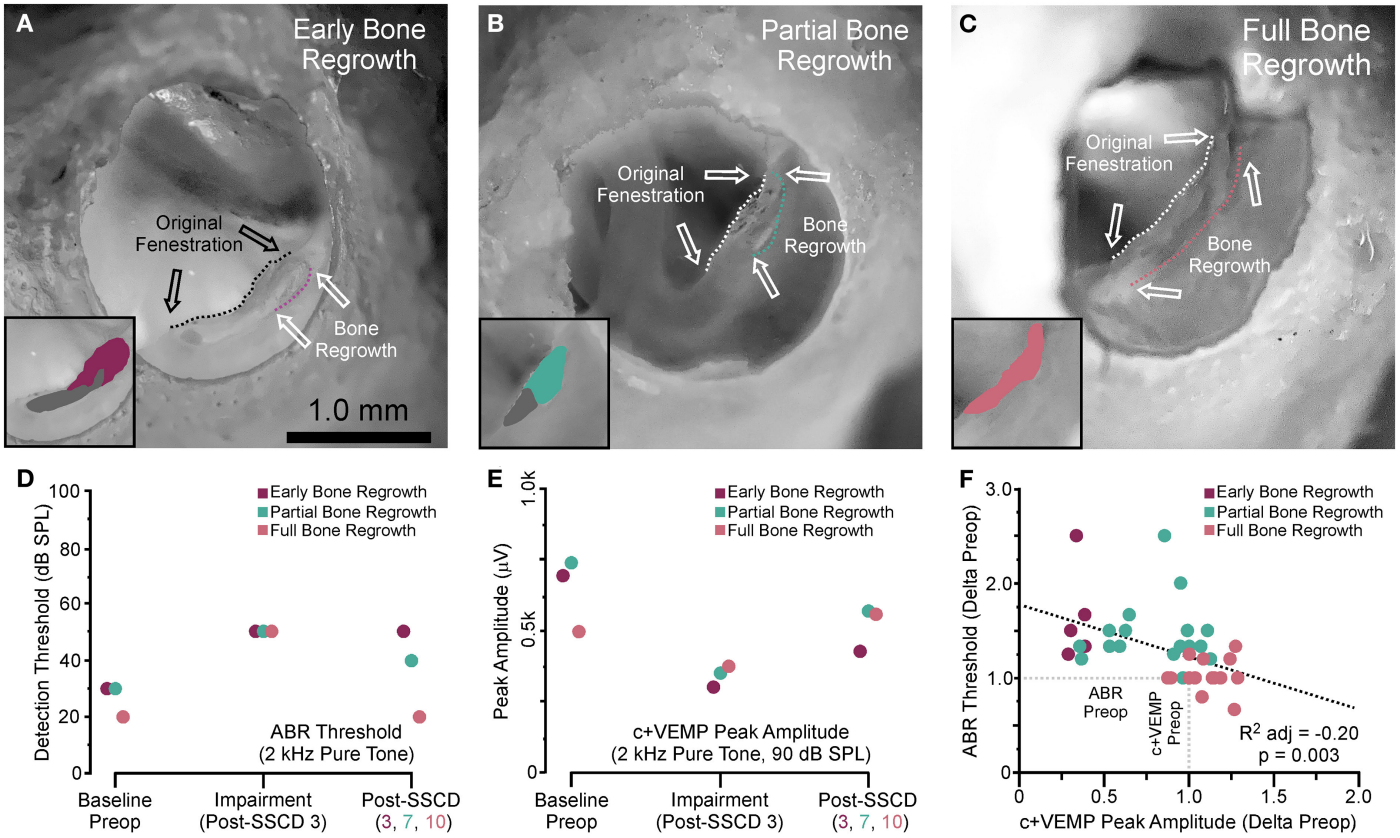
Photomicrograph correlation between ABR and c+VEMP thresholds/amplitudes at experimental endpoints and indicators of bone regrowth. **(A)** Photomicrograph showing early bone regrowth 3 days after SSCD. **(B)** Photomicrograph showing partial bone regrowth 7 days after SSCD. **(C)** Photomicrograph showing full bone regrowth 10 days after SSCD. **(D)** Chart showing the changes to ABR thresholds at 2 kHz for the animals shown in **(A–C)** at baseline, 3 days post-SSCD (impairment), and at the experimental endpoint (post-SSCD days 5–10). **(E)** Chart showing the changes to c+VEMP peak amplitudes at 90 dB SPL for the animals shown in **(A–C)** at baseline, 3 days post-SSCD (impairment), and at the experimental endpoint (post-SSCD days 5–10). **(F)** Scatterplot showing a negative correlation between ABR and c+VEMP based on bone regrowth status.

After both large and small SSCDs, we saw a trend in the changes to ABR and c+VEMPs wherein an impairment would emerge that was followed by a reversal of this impairment toward the baseline, i.e., preoperative, levels. This recovery in both groups suggested a conserved mechanism at work within both groups. As the major difference between the groups is the size of the fenestration we hypothesized that just like humans undergoing the old fenestration operations for otosclerosis, the site of bony dehiscence regrows into and obliterates the canal if the fenestration is too small or bone debris is left at the site of the fenestration (26–30). This is in contrast to patients who require direct surgical plugging of the SSCD to resolve the third window symptoms (14, 15, 21). To investigate this hypothesis, we carried out another acute experiment with eight animals (4M, 4F), where the experimental endpoint was the correlation of ABR and c+VEMP measurements with corresponding brightfield stereomicroscope evidence of bone regrowth. For all animals, the left ear was dissected, and the fenestration site was imaged with a condenser brightfield microscopy (Figure 5). Figure 5A shows a representative example of an animal with limited bone regrowth early after SSCD (post-SSCD day 3), with comparison to an animal with partial (post-SSCD day 7, Figure 5B) and full (post-SSCD day 10, Figure 5C) bone regrowth. Figures 5D, E show the ABR and c+VEMP data from the three animals shown in Figures 5A–C (Early. Partial, and Full Bone Regrowth, respectively). Illustrated is the SSCD-induced impairment and the data collected on the experimental endpoint (post-SSCD day 3, 7, or 10). Finally, Figure 5F shows the correlation between ABR and c+VEMPs as a function of the state of bone regrowth. This analysis suggests a strong correlation between bone regrowth and “recovery” of the physiological function of the inner ear [ABR Threshold (dB) = 203–0.84^*^c+VEMP Amplitude (μV), adjusted *R*^2^ = 0.29, *p* < 0.001].

### Micro-CT scanning confirms that bone resurfacing and not canal obliteration is associated with bone regrowth

**Figure 6:**
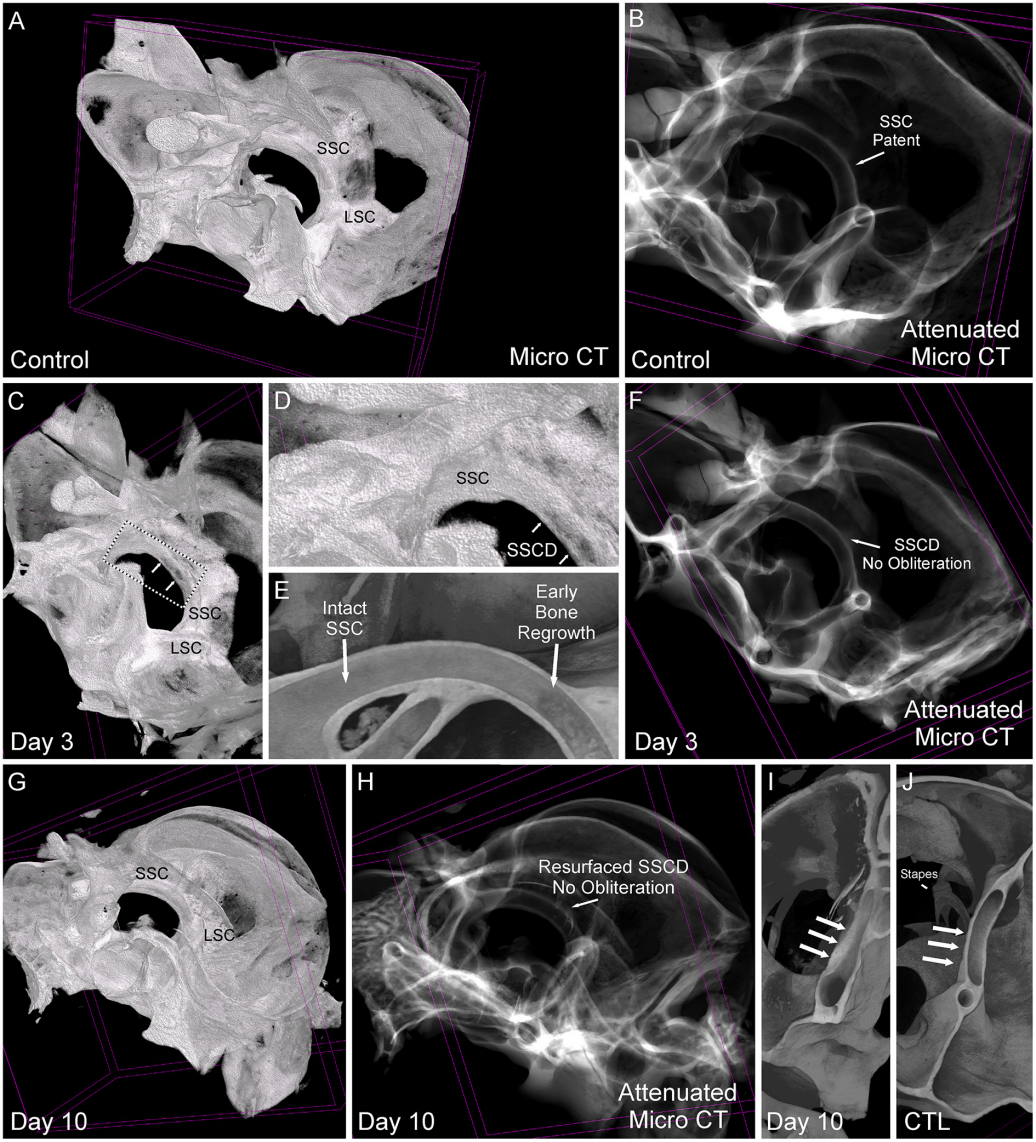
Comparative micro-CT analysis of control and post-SSCD day 3 and day 10. **(A)** Micro-CT scan of left control **(A, B, J)**, day 3 post-superior semicircular canal dehiscence (SSCD) **(C–F)**, and day 10 post-SSCD **(G–I)** after small (1 mm) SSCD surgery. **(A)** Micro-CT maximum intensity projections (MIP) with a 3D rendering of control left inner ear. Note the normal intact superior semicircular canal (SSC) and lateral semicircular canal (LSC). **(B)** Micro-CT with attenuated images shows that the lumen of the SSC is naturally patent. **(C)** Micro-CT MIP with 3D rendering shows the day 3 post-SSCD site of a small (1 mm) SSCD (double arrows). Note the position of the SSC and LSC. A bounding box containing the SSCD (double arrows) is shown in higher magnification in **(D)**. **(D)** The SSC with the beginning of osteoneogenesis within the SSCD can be seen (double arrows). **(E)** The micro-CT view inside of the intact SSC (left) reveals the adjacent early bone regrowth (right) at day 3 post-SSCD. **(F)** Micro-CT with attenuated images shows that the lumen remains patent at day 3 post-SSCD. **(G)** Micro-CT MIP with 3D rendering of a small (1 mm) SSCD on postoperative day 10. Note the positions of the SSC and LSC. **(H)** Micro-CT with attenuated images shows that the lumen remains patent and without bony obliteration, as is the normal condition shown in **(B)**. **(I)** Micro-CT view inside the left SSC on day 10 post-SSCD [small (1 mm) fenestration]. Note the increased thickness of the resurfaced SSC bone (triple arrows) compared to the control SSC has shown in **(J)**. **(I)** Micro-CT inside the left SSC shows that the natural SSC has uniform wall thickness (triple arrows) that is thinner than the resurfaced/regrown SSC. CTL, control.

Based on the stereomicroscope, it was unclear whether the bone regrowth was accompanied by obliteration of the lumen, or if the bone resurfaced to form an intact canal. Therefore, we took the samples used in Figure 5 and completed micro-CT scans to visualize the site of surgically created SSCD and determine the outcome of bone regrowth (Figure 6). By comparing a control specimen (Figures 6A, B, I), the day 3 specimen (b), and the day 10 specimen (Figures 6G, H, J), it is clear that the bone resurfaces does not obliterate the canal. However, it does regrow as thicker bone (Figures 6H, I). Thus, unlike the most common surgical method in patients with SSCD, which plugs the dehiscence, our gerbil model repairs the SSCD by resurfacing the canal with qualitatively thicker bone. Hence, a second surgical procedure to plug the defect is unnecessary.

## Discussion

For patients who are clinically suspected of having SSCD, there are three essential diagnostic features that are typically present: (1) high-resolution temporal bone CT demonstration of the dehiscence; (2) cVEMP increased amplitude and decreased threshold on the side of the SSCD; and (3) pseudoconductive hearing loss. In patients whose symptoms are severe enough to warrant surgical intervention, the most common way of accomplishing resolution of the SSCD and associated symptoms is a middle cranial fossa craniotomy and plugging of the SSCD. For an ideal model of this disorder, all of these features should be present, and resolution should follow the plugging of the SSCD.

### Imaging

In our gerbil model, we used microsurgical techniques to create and measure the SSCD; therefore, CT imaging was not necessary. However, we used micro-CT to better understand the nature of the osteoneogenesis resulting in the reconstitution of the SSC (Figure 6).

### c+VEMP

It was over a century ago that Robert Bárány began using caloric irrigation and his vertical axis rotational chair to assess horizontal canal function, yet it was not until 1994 that Colebatch and colleagues developed the sound-evoked cervical vestibular evoked myogenic potential (cVEMP) (31) to study the gravitational receptors. Sound-induced activation of the saccule leads to an inhibition of the sternocleidomastoid muscle, and this inhibitory potential can be recorded as the cVEMP [for review see (31–35)]. The evoked potentials recorded from a number of other muscles have been studied as well; however, it is the sternocleidomastoid muscle that is most consistently used in research and clinical applications. It has also been shown that both ipsilateral and contralateral cVEMP can be recorded from the sternocleidomastoid muscle following ipsilateral stimulation (31–39). Bone-conducted stimuli have also been used to evoke cVEMP responses [for review see (33, 35)]. All of these cVEMP methods depend on voluntary contraction of the sternocleidomastoid muscle so that the evoked inhibitory potential can be measured, which is not possible in an anesthetized animal.

For a person without a mobile third window, the normal cVEMP threshold is 95 dB nHL. For patients with SSCD, there is typically an increased cVEMP amplitude and reduced cVEMP threshold to 81 ± 9 dB nHL (9); however, the threshold may be reduced as low as 60 to 65 dB nHL. In a small number of SSCD patients, particularly older patients, a cVEMP response cannot be measured. As outlined in the Introduction, it is not possible to record a cVEMP response in anesthetized animals since the voluntary contraction of the sternocleidomastoid muscle required to record the relaxation potential associated with sound-induced saccular stimulation cannot be accomplished. We have demonstrated that the c+VEMP response can be recorded in the gerbil and that the increased amplitude of the response after the creation of SSCD was observed. The c+VEMP was elicited at lower stimulus intensities than patients, and we were unable to measure the threshold as it was too low.

In patients with SSCD and other sites of dehiscence resulting in third window syndrome, cVEMPs are useful diagnostic indicators, with patients exhibiting abnormal responses to auditory clicks or tone bursts used in this test (7, 14–16, 36, 37). The cVEMP amplitudes in the affected labyrinth are increased, and thresholds are reduced (7, 9, 14–16, 34, 40). After surgical plugging of the SSCD, cVEMP thresholds and amplitudes were normalized (40). An ideal animal model of SSCD would show elevated amplitude responses and reduced thresholds in response to auditory stimulation of the sacculus that would normalize with the closure of the SSCD. A limitation of earlier animal models was the attempt to record cVEMP responses from the sternocleidomastoid muscle. The sound-induced VEMP is inhibitory for neck flexor muscles and excitatory in neck extensor muscles (13). Thus, it is required to have the SCM contracted to record the inhibitory relaxation potential, which is not possible in an anesthetized animal. However, we took advantage of the excitatory responses in the extensor muscles to record what we term c+VEMPs. In humans, the normal threshold for cVEMP is 95 dB HL. For gerbils, the c+VEMP threshold is 45 dB HL, so measuring a reduced threshold was not possible. However, just as in patients with SSCD, we recorded increased amplitude of the c+VEMP response in our surgically created large (2 mm) SSCD animals that remained elevated for 1–2 weeks before the amplitude returned to normal, in the same animal, with the closure of the SSCD *via* osteoneogenesis.

While not termed c+VEMPs, other investigators have studied the excitatory sound-induced VEMP recorded from the splenius capitus muscle of humans (41–44). This response from neck extensor muscles had not previously been reported in an animal model of SSCD but has been studied in multiple species for different experimental purposes.

Yang et al. used neck extensor muscle cVEMPs in a guinea pig model to monitor the vestibulotoxicity of gentamicin otic drops (45). The compound 3,3′-iminodiproprionitrile (IDPN) has been reported to be neurotoxic and vestibulotoxic resulting in loss of vestibular sensory epithelial cells in rats and mice and leading to irreversible loss of peripheral vestibular function (46). Negishi-Oshino et al. used neck extensor muscle cVEMPs, in addition to behavioral rotarod, beam crossing, and air-righting reflex tests in adult C57BL/6J mice to measure IDPN vestibulotoxicity and thereby create an *in vivo* model of adult mammalian vestibular dysfunction (47). Finally, neck extensor muscle VEMPs have been studied in mini-pigs and rats (48, 49).

On post-SSCD day 1 in the small (1 mm) group, there was a small increase in latency, and the amplitude of the c+VEMP response was greatly decreased along with an increase in the stretch reflex. These observations are consistent with acute otolithic dysfunction (50). Dyball et al. reported that, in normal humans, tapping the forehead delivering bone-conduction stimuli produces a cVEMP response and that the stretch receptors can also be activated by skull taps resulting in a later potential associated with the stretch reflex (58). They also found that patients with unilateral vestibular loss had larger late peaks on the affected than the normal side which in the context of our findings suggests these animals experienced transient acute otolithic dysfunction. As will be described shortly, this short-lived loss of c+VEMP function was not seen in the ABR data. Based upon the data reported herein, in all future experiments, the animal model will use 2-mm SSCD, and 1-mm SSCD will not be utilized.

### Auditory brainstem response

Attias et al. (51) used bone-conduction ABR and air-conduction ABR to measure the pseudoconductive air-bone gap resulting from the surgical creation of a 0.6-mm SSCD in the sand rat. Attia et al. showed “significant deterioration of the air-conduction thresholds to clicks (*t*_9_ = 18.4, *p* < 0.001) and tone bursts (*t*_9_ = 6.5, *p* < 0.001) in the absence of a significant change in bone-conduction thresholds.” This means that altered air-conducted ABR thresholds explained the increased air-bone gap in their model. We followed their established precedent and, therefore, we did not use a bone-conduction ABR approach. Delsmann et al. (52) used the same approach that we used in recording ear-specific air-conduction ABR as a proxy to measure the conductive hearing loss in the *Hyp* mouse model of X-linked hypophosphatemia caused by hypomineralization of the auditory ossicles.

For our data, we found that for the large SSCD group, while the data were variable, there was a recovery of ABR thresholds as the SSCD closed, suggesting that surgical disruption of cochlear function was not due to hair cell loss and irreversible hearing loss and that the spontaneous bone regrowth and closure of the SSCD obviate the need of a second surgical procedure to surgically plug the SSCD. This not only avoids unnecessary animal surgeries but also allows the reversal of the SSCD and recovery of function in a more holistic way.

### Evidence of bone regrowth and physiological return to baseline

The regrowth of bone to close the surgically created SSCD and reverse the physiologic dysfunction without a second surgical procedure is another strength of our animal model. The micro-CT data showed that the defect heals by resurfacing rather than bony obliteration of the canal (Figure 6). Initially, we did not expect the observed differences in the small vs. large SSCD cohorts, or the reversal of the c+VEMP and ABR findings over time. However, we reviewed the literature regarding an operation no longer performed in patients, the one-stage fenestration surgery for otosclerosis, to gain insight into our experimental observations. With otosclerosis, the stapes footplate becomes fused to the otic capsule effectively dropping from the physiologically necessary two mobile windows down to one. The lateral semicircular canal fenestration operation was developed to create a new second mobile window to improve the conductive hearing loss these patients experienced. Although many others contributed to the development of the fenestration procedure, Julius Lempert was the first to develop the one-stage fenestration operation (26–30). As the technique evolved, two critical issues emerged related to preventing bone regrowth and closure of the new surgically created second mobile window: (1) avoiding a fenestration that was too small and (2) removing the bone dust from the area surrounding the fenestration (27, 28, 30). Dewyer et al. reported the temporal bone pathology of a woman who had bilateral fenestration operations performed for otosclerosis (30). On the left side, there was histologic evidence of osteoneogenesis within the fibrous tissue sealing the lateral semicircular canal fenestration. In the context of our new animal model, the small SSCD proved to be too small and facilitated early closure of the fenestration. Because of the small diameter of the gerbil SSC and the associated surface tension between the surgically exposed perilymph, surrounding bone, and bone dust, it is very difficult to wash the bone dust away from the SSCD. For these reasons, in future experiments, we will use gerbils with large (2 mm) SSCDs for our longer-term behavioral studies.

Normally, the endochondral bone of the otic capsule calcifies in its adult conformation and does not undergo remodeling (53). Osteoblast–osteoclast bone remodeling units have not been normally seen in normal otic capsule bone (54). The bone lining cells of mammals form a membrane-like layer physically partitioning the bone matrix from the extracellular fluid, which in the cochlea, semicircular canals, and rest of the labyrinth, is perilymph. Chole and Tinling (55, 56) reported that in gerbil endosteal cochlea there was a relatively high ratio of bone lining cells to bone surface of 71.1 ± 15.1 (ulna was 100). However, they did not study the superior semicircular canal. The bone lining cells are thought to play an important role in the recruitment of osteoclasts and in turn osteoneogenesis to a local site after injury, in our animals by the surgical creation of the SSCD, by exposing the surface of the bone that initiates a signal for the chemoattraction of osteoclasts and/or their progenitors (55, 56). There are other well-known examples of injuries of the otic capsule that result in osteoneogenesis including transverse temporal bone fractures and cochleostomy with the placement of a cochlear implant electrode array. Nager (57) histologically demonstrated that the entire superior semicircular canal can become obliterated by bone by osteoneogenesis induced by a transverse temporal bone fracture. Surgical cochleostomy for cochlear implant electrode insertion in humans and animal models can induce osteoneogenesis. Quesnel et al. (58) reported postmortem histopathologic evidence of osteoneogenesis after cochleostomies, in an anterior–inferior location relative to the round window, and placement of bilateral hybrid electro-acoustic stimulation cochlear implants. In an experimental guinea pig cochlear implant model, O'Leary et al. (59) reported cochleostomy and electrode insertion-induced osteoneogenesis. In a subsequent study, using the same animal model, they found that systemic dexamethasone (a glucocorticosteroid) reduced this osteoneogenesis (60). This later observation may delay the time of bony obliteration in our SSCD model which would extend the time available for additional experimental studies of the sequelae, particularly with cognitive dysfunction and sound-induced vestibular dysfunction, seen in patients with SSCD.

Future studies will focus on the histopathologic response to the surgical creation of the SSCD in our model and the basic mechanisms associated with this osteoneogenesis.

### Evidence for persistent changes to cochlear and vestibular function after c+VEMP amplitudes and abr thresholds return to baseline

#### ABR thresholds and wave morphology

There was strong evidence for the correlation between the closure of the SSCD by osteoneogenesis and the resolution of cochlear and vestibular impairments; however, deeper analysis of the data suggested that persistent changes can remain after ABR thresholds have returned to preoperative-like (non-significant difference) levels (see Supplementary Figures 2–4; Figure 3B). Figure 4 shows that latency to ABR wave I and the 7 peaks (waves I–VII) associated with activation of ascending central auditory neuraxis was persistently altered by the surgical creation of and/or physiologic changes associated with the SSCD. Figure 4A shows a significant effect of superior semicircular canal fenestration on wave I ABR latency at 2 kHz and a persistently altered c+VEMP N1 latency (preoperative vs. recovered latency). Together, these data demonstrate that despite resurfacing of the SSCD and return of ABR thresholds and amplitudes as well as c+VEMP amplitudes, significant changes to the timing of nerve activation as well as the relative strength of central auditory neuraxis activation persist after recovery.

In patients with SSCD who have their superior semicircular canal plugged by either the middle cranial fossa approach or the transmastoid approach, persistent pseudoconductive hearing loss is well-known, but not understood (23–25). In our animal model, an inspection of the number of visible ABR peaks, which signify activation along with the neuraxis [cochlear nucleus (CN), superior olivary complex (SOC), inferior colliculus (IC), medial geniculate nucleus (MGN), and auditory cortex] (see Figure 2A) at each decibel stimulus level (90 db SPL to 20 dB SPL) showed a significant decrease in auditory neuraxis potentials evoked at each stimulus level compared to preoperative ABR waveforms. In the preoperative waveforms, there was increased activation at peaks II through VII compared to each experimental endpoint. Thus, the preoperative 40 dB SPL 2 kHz stimulus activates higher up the auditory neuraxis than 40 dB SPL 2 kHz stimulus in the same animal after the: (1) SSCD has closed by osteoneogenesis; and (2) ABR and c+VEMPs measures returned to a non-significant difference from preoperative levels. The medial geniculate nucleus and auditory cortex activation ABR potentials seem to be affected the most. This might drive the persistent hearing loss if the thalamus and the cortex have diminished activation at the same dB SPL stimulus level. Together, these could suggest that there are persistent changes in both peripheral function due to hair cell injury/dysfunction (e.g., latency) and central (ABR waveforms) or even that central circuit plasticity has altered signal propagation/amplification, which has altered some aspects of cochlear physiology (e.g., efferent system) after short-term diversion of sound pressure flow to the third mobile window (i.e., SSCD).

The noise produced by a surgical drill is a confounding factor in the interpretation of transient and persistent hearing loss after the creation of an experimental dehiscence. From an otologic drill perspective, both a temporal craniotomy (middle cranial fossa) approach and a transmastoid plugging of an SSCD are comparable clinical procedures that can result in postoperative high-frequency sensorineural hearing loss. Ward et al. reported the hearing outcomes after surgical plugging of SSCD patients using a middle cranial fossa approach and found that a mild high-frequency sensorineural hearing loss was present and persisted in 25% but there was no change in speech discrimination (24). A similar experience was reported by Ellsperman et al. after surgical plugging of SSCD patients using either a middle cranial fossa approach or a transmastoid approach (25). Both approaches could produce high-frequency hearing loss (8 kHz) because the surgical drill delivers high-frequency acoustic energy to the cochlea *via* the temporal bone. Since it is well-known the temporal bone dissection with contemporary microsurgical drills can result in high-frequency sensorineural hearing loss (61), it is not surprising that persistent hearing loss, measured by ABR, was found in our animal model at 16 kHz. However, drill noise alone cannot explain the greater ABR threshold elevation for the small dehiscence group (relative to the large dehiscence group) at 7 days post-SSCD, given the longer drill exposure in the latter group.

#### c+VEMP amplitudes

Initially, an increase in latency was observed in N1 of the c+VEMP for both small and large SSCD; however, there were no significant differences between the large and small SSCD c+VEMP latency after recovery. In both the small and large SSCD cohorts, the elevated c+VEMP amplitudes returned to preoperative-like (non-significant difference) levels (see Supplementary Figures 2G–L, 3C; Figure 3C), just as they do in patients with SSCD after plugging the dehiscence (40). Welgambola et al. studied cVEMP responses of 20 normal volunteers, 10 newly diagnosed subjects with SSCD, and 12 subjects who underwent successful plugging of their SSCD using a middle cranial fossa approach (40). In the subjects who had to plug their SSCD, the thresholds for evoking cVEMPs using air-conducted tones were pathologically lowered, and the amplitudes were elevated. Successful canal plugging resulted in normal cVEMP thresholds and reduced amplitudes. We were unable to measure reduced thresholds of the c+VEMP response in gerbils because of their naturally low c+VEMP thresholds; however, the elevated c+VEMP amplitudes returned to preoperative-like (non-significant difference) levels (Supplementary Figures 2G–L, 3C; Figure 3C) after the closure of the SSCD by osteoneogenesis (see Figures 5, 6).

## Conclusion

The large (2 mm) SSCD used in our gerbil model resulted in electrophysiologic findings that mirror findings in patients with SSCD. Our animal model improves upon the fidelity to the human findings from previously reported models (10, 11) and provides a more detailed view of the healing process and associated physiological findings. The advances are in several areas. First, although the earlier studies reported elevated ABR thresholds after the experimental SSCD-induced elevations to ABR thresholds, we have provided evidence that effects differ markedly with SSCD size and document timelines of impairment and recovery. Second, we have added a functional vestibular measure that showed correlated impairment, recovery, and persistence between cochlear and vestibular physiology. This is a key feature that will allow a detailed study of the functional connectivity between vestibular and cochlear input/output at the peripheral and central levels. Third, this animal model has been developed explicitly for compatibility with a wide range of behavioral paradigms to test cognitive and perceptual impairments as a function of SSCD status (impairment/recovery). Finally, we have documented that the SSCD-related changes recovered to baseline as the defect heals *in situ* by bony resurfacing of the SSCD without obliteration. Hence, there is no need for a second surgical procedure to plug the SSCD. Because peripheral vestibular disorders and associated asymmetric input to the central nervous system have been reported to provide a unique window to study cognitive dysfunction (62), this SSCD model will provide the opportunity to perform the behavioral as well as electrophysiological, cell, and molecular biology studies that are needed to understand the cognitive dysfunction seen in patients with SSCD and other vestibular disorders.

## Data availability statement

The datasets are available upon reasonable request and with permission of the Institutional Animal Care and Use Committee.

## Ethics statement

The animal study was reviewed and approved by the Rutgers University IACUC (research protocol PROTO202000179).

## Author contributions

PAW and TMM contributed to the conception, design of the study, and wrote the first draft of the manuscript. TMM organized the database and performed the statistical analysis. OJVO, BTM, PAW, M-AT, VLS, SD, JDG, CDB, and TMM wrote sections of the manuscript. All authors contributed to manuscript revision, read, and approved the submitted version.
