# Supplementary material for: New model of superior semicircular canal dehiscence with reversible diagnostic findings characteristic of patients with the disorder
Source: Front Neurol. 2023 Jan 19;13:1035478. doi: 10.3389/fneur.2022.1035478 (PMC9892720; doi:10.3389/fneur.2022.1035478)
Supplement: Supplementary file 1 [file Data_Sheet_1.pdf]

Supplementary Materials

Supplementary Results

Tracking Acute Changes to Peripheral Impairment After Small Superior Semicircular Canal Dehiscence

To establish a timeline for the changes to ABR thresholds and c+VEMP amplitudes that occur after SSCD we carried out acute recordings over 10 days in animals (N=5; 2M, 3F) that received the small (1 mm) fenestration (Supplementary Fig. 1). For each animal in the small SSCD group, recordings were carried out prior to surgery and 1, 3, 5, 7 and 10 days after microsurgical creation of the SSCD. Analysis was carried out within animals and between animals across days. There were significant main effects indicating differences in the air-induced ABR thresholds between frequencies ($F[df=2.8,11.2]=35.7$, $p<0.001$, Greenhouse-Geisser adjustment) and over time after creation of the SSCD ($F[df=1.3,5.3]=11.4$, $p=0.014$, Greenhouse-Geisser adjustment). Least significant differences (LSD) multiple range tests were used to make direct comparisons between pre-SSCD thresholds at each frequency (baseline) and the various day by day threshold changes post-SSCD (Supplementary Figs. 2B-F). For the mean ABR threshold values across the 5 frequencies (assessed in 10 dB SPL increments), the ABR thresholds were elevated from baseline on postoperative days 1 ($p<0.01$), 3 ($p<0.01$), 5 ($p<0.01$) and 7 ($p<0.05$). This significant threshold elevation disappeared by post-SSCD day 10. However, the time course of threshold changes varied with frequency (Supplementary Fig. 3B). At 1, 2, 4 and 8 kHz, there were significant threshold increases on day 1, day 3 and day 5 ($p<0.05$) after the surgery. At 16 kHz, the duration of the significant threshold elevation extended to 7 and 10 days after the surgery ($p<0.05$). Table 1A in the published manuscript shows the means and SEMs and significance tests for ABR thresholds in each individual animal that received small SSCD fenestrations.

In the same animals c+VEMPs were measured consecutive to ABRs on each post-SSCD day (Supplementary Figs. 2G-L and 3C). Preoperatively, the c+VEMP amplitudes increased significantly at 95 dB SPL relative to 80 dB SPL ($p<0.05$) and maintained that level at 100 dB SPL ($p<0.05$ relative to 80dB SPL). Repeated-measures ANOVA across intensities and post-SSCD sessions showed a significant main effect indicating differences as a function of sound intensity in the c+VEMPs ($F[df=2.69,10.78]=5.174$, $p<0.05$, Greenhouse-Geisser adjustment). The main effect over time after creation of the SSCD was not significant; individual animal responses are shown in Supplementary Figures 2G-L. LSD multiple range tests were used to make direct comparisons between pre-SSCD thresholds at each intensity (baseline) and the day-by-day threshold changes post-SSCD (Supplementary Fig. 3C). The mean pooled c+VEMP magnitudes across the 5 intensities were reduced significantly from baseline on only postoperative day 1 ($p<0.001$). This initial reduction was followed by a return to values that did not differ significantly from baseline. Table 1B in the published manuscript shows the means and SEMs and significance tests for c+VEMP amplitudes in each individual animal that received small SSCD fenestrations.

Closer examination showed that the significant c+VEMP effects were detected for the longest time at the most intense stimulus intensity (100 dB SPL). At 80 dB SPL and 85 dB SPL, there was a significant reduction from the preoperative amplitude at only at post-SSCD day 1 ($p<0.001$). The reduction persisted longer for 90 dB SPL stimulation, where it appeared on post-SSCD days 1 ($p<0.01$), 3 ($p<0.05$) and 7 ($p<0.05$). The reduction (relative to preoperative measures) was also noted at 95 dB on post-SSCD days 1 ($p<0.01$) and 3 ($p<0.05$) and at 100 dB on post-SSCD days 1 ($p<0.001$), 3 ($p<0.05$), 5 ($p<0.05$) and 7 ($p<0.05$).

Because the c+VEMP data were elicited by 2 kHz induced tones, the relationship between the ABR amplitudes at 2 kHz and the c+VEMP amplitudes were explored with regression analysis (Supplementary Fig. 3D). Results shown are based on the 2 kHz data in Supplementary Figures 2B-F

and the 90 dB SPL c+VEMP data in Supplementary Figure 2G-L. For this analysis ratios were calculated to show changes above (>1.0) or below (<1.0) preoperative baselines. The relationship is significant (c+VEMP [μV] = 3.008-1.67*ABR Threshold [dB], adjusted R^2 = 0.42, $p<0.001$). This tight correlation suggested a physiological coupling between the effects on sensory (ABR) and motor (c+VEMP) consequences of the small SCCD, likely of inner ear origin. Further analysis measured the correlation between c+VEMP amplitudes at 90 dB SPL and ABR amplitudes at 90 dB SPL for 2 kHz and 16 kHz frequency measurements in Supplementary Figure 2. For the 2 kHz correlation (Supplementary Fig. 3E, left) there was a significant relationship (c+VEMP [μV] = -2.47+0.46*ABR Amplitude [μV], adjusted R^2 = 0.35, $p<0.001$). The 16 kHz correlation (Supplementary Fig. 3E, right) did not show a significant relationship between c+VEMP amplitude and ABR amplitude at 90 dB SPL (c+VEMP [μV] = 327+0.24*ABR Amplitude [μV], adjusted R^2 = 0.054, p=0.11).

Effects of Dehiscence Size on ABR and c+VEMP Physiological Properties.
A direct comparison of the effects of the small versus large dehiscence on post-SSCD day 7 clearly shows the polarizing effect of SSCD size on c+VEMP amplitude (Supplementary Fig. 5A). Mixed model ANOVA of the ABR threshold data (between groups factor: SCCD size, within groups: test days and frequency) showed significant main effects of dehiscence size (F[1, 8]=14.40, $p<0.001$), test day (F[df=2.69,10.78]=7.08,$p<0.05$, Greenhouse-Geisser adjustment), frequency (F[df=2.48,19.81]=36.68,$p<0.001$, Greenhouse-Geisser adjustment) and a significant SSCD size by test day interaction(F[df=1.00,8.00] =13.66, $p<0.01$, Greenhouse-Geisser adjustment). LSD multiple range tests revealed no significant threshold differences preoperatively, but the thresholds were elevated for the small SSCD relative to the large SSCD subjects at all frequencies on post-SSCD day 7 ($p<0.05$ at each frequency).

Next, we compared ABR amplitude and its corresponding ABR latency as well as c+VEMP amplitude and latency by SSCD size (Supplementary Fig. 5B-C). For ABR data, only those amplitudes and latencies that were present for every animal were included in the regression analyses (90 dB, 80 dB and 70 dB SPL). This analysis shows that ABR amplitudes between 90 and 70 dB SPL on post-SSCD day 7 have a significant increase in ABR amplitudes for large SSCD animals compared to small SSCD animals at 2 kHz stimulation (F[1, 8]=15.75, $p<0.01$). Analysis of ABR latency for 2 kHz (F[1, 8]=4.97, p=0.056) shows no significant effect of SSCD size on ABR latency at post-SSCD day 7. Comparison of c+VEMP amplitudes and their corresponding latencies between small and large SSCD on day 7 at all stimulus levels (100 to 80 dB SPL) show a significant increase in c+VEMP amplitude for large SSCD compared to small SSCD animals (F[1, 8]=21.51, $p<0.001$); and no significant difference in c+VEMP latencies between SSCD small and large animals on this post-SSCD day (right, F[1, 8]=1.43, p=0.26). While there were no significant differences between ABR or c+VEMP latency based on SSCD size, there was a significant relationship between amplitudes and latency for ABRs (e.g., Supplementary Fig. 5D; ABR Amplitude [μV] = 2524-562*ABR latency [ms], adjusted R^2 = 0.23, $p<0.001$) and c+VEMPs (c+VEMP Amplitude [μV] = 1455-214*c+VEMP latency [ms], adjusted R^2 = 0.21, $p<0.001$).

The differential effects of fenestration size are also apparent in the relationships between the ABR c+VEMP amplitude at 90 dB SPL and ABR thresholds for 2 kHz (Supplemental Fig. 5E). Here you can see a clear bifurcation of the c+VEMP amplitudes at thresholds of 40 dB SPL and greater for 2 kHz. Dividing the data by the bifurcation lines allowed us to measure correlation differences between thresholds above and below these lines. In Supplemental Figure 5F you can see that at 2 kHz correlations below the 40 dB SPL threshold are not significant (c+VEMP [μV] = 563+0.13*ABR Amplitude [μV], adjusted R^2 = -0.02, $p<0.001$); however, there is a strong correlation between c+VEMP amplitudes and ABR amplitudes at thresholds above 40 dB SPL (c+VEMP [μV] = -8.01+1.18*ABR Amplitude [μV], adjusted R^2 = 0.60, $p<0.0001$).The significant correlations occur on those days when we see the significant shifts in both c+VEMP amplitude and ABR threshold for both small (see Supplementary Fig. 2 and Supplementary Fig. 3) and large (see Supplementary Fig. 4 and

Fig. 2) SSCDs, where preoperative measures and endpoint measures are similar and thus not correlated. This suggested that some regenerative process such as osteoneogenesis at the fenestration site could be driving the return of the physiological measures towards the preoperative baseline responses.

Supplementary Figures

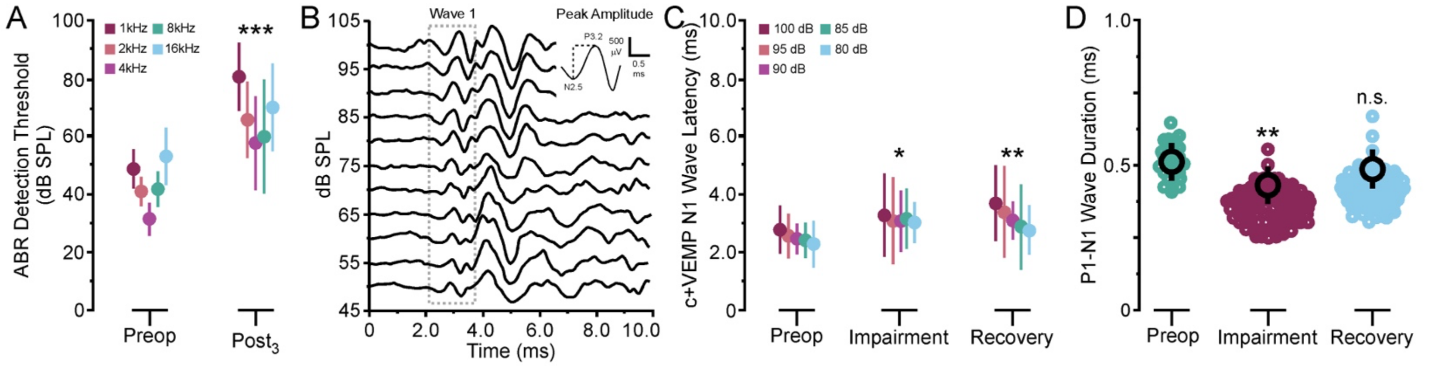

Supplementary Figure 1. Physiological methods for establishing vestibular dysfunction after SSCD. A) Line chart showing the ABR thresholds for 1, 2, 4, 8, and 16 kHz preoperatively and 3 days post-SSCD. B) Representative example of a c+VEMP response induced by a 2 kHz tone at 100 to 50 dB SPL. Inset: A typical wave 1 example where c+VEMP amplitudes are measured by subtracting the negative N wave 1 from the positive P wave 1. C) Line chart showing the latency to P wave 1 for all animals at baseline (preoperative), during impairment (small SSCD: post-SSCD day 1, post-SSCD day 3; large SSCD: post-SSCD day 7, post-SSCD day 14), or during recovery towards baseline (small SSCD: post-SSCD day 5, post-SSCD day 7, post-SSCD day 10; large SSCD: post-SSCD day 21, post-SSCD day 28). D) Chart showing the change in wave 1 duration for all animals at baseline (preoperative), during impairment (small SSCD: post-SSCD day 1, post-SSCD day 3; large SSCD: post-SSCD day 7, post-SSCD day 14), or during recovery towards baseline (small SSCD: post-SSCD day 5, post-SSCD day 7, post-SSCD day 10; large SSCD: post-SSCD day 21, post-SSCD day 28). Circles are means with SEMs (lines). *$p<0.05$, **$p<0.001$, ***$p<0.0001$.

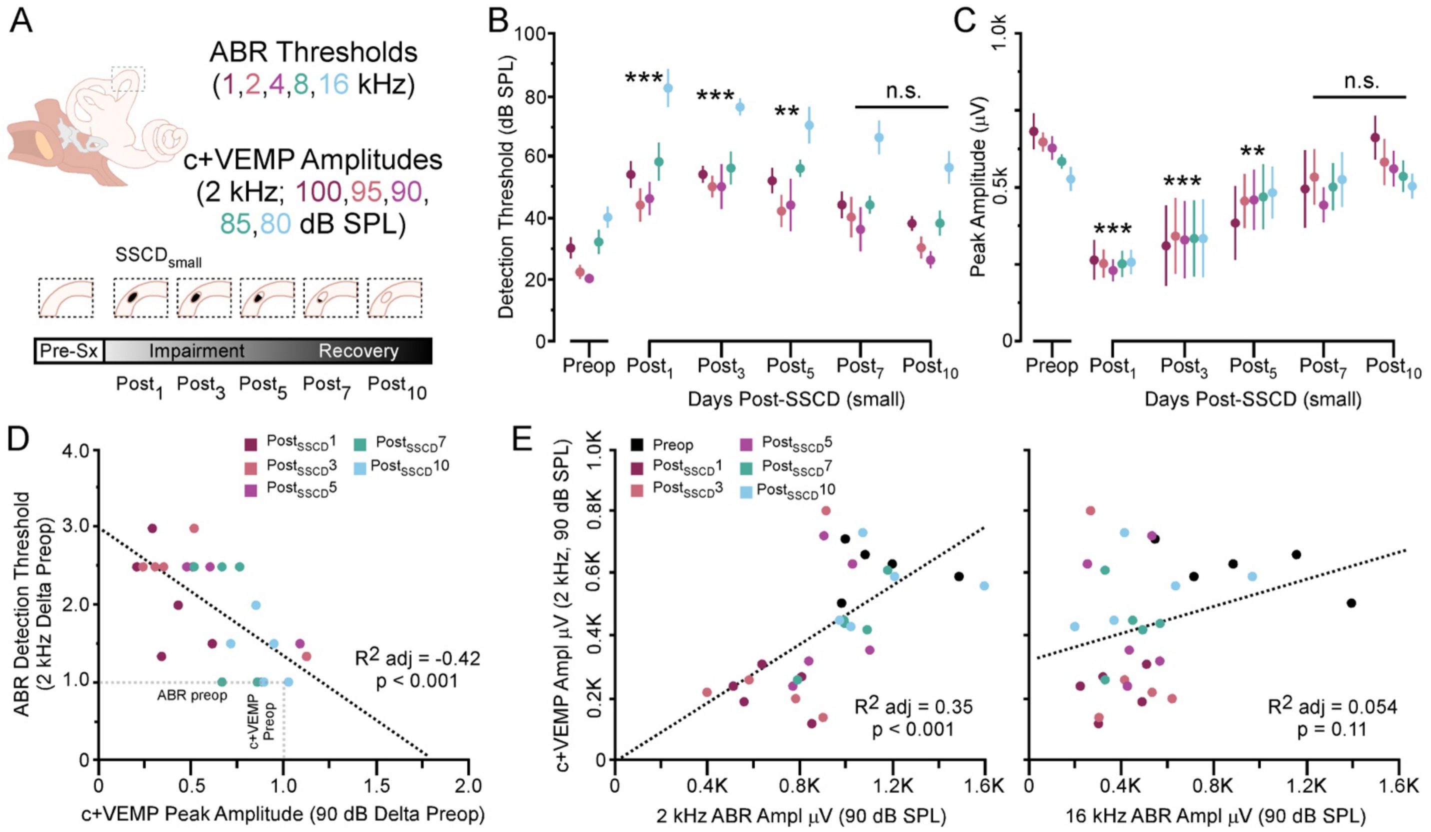

Supplementary Figure 2. The correlated group changes to sound-induced ABR thresholds and c+VEMP amplitudes after small SSCD. A) Diagram showing the theoretical recovery of ABR thresholds and c+VEMP amplitudes over days (post-SSCD days 1, 3, 5, 7, and 10). B) Line chart shows the group means for ABR thresholds over postoperative days in animals that received the small SSCD. C) Line chart shows the group means for c+VEMP amplitudes over post-SSCD days in animals that received the small SSCD. D) Scatterplot showing a significant negative correlation between auditory thresholds and c+VEMP amplitudes throughout impairment and recovery from small SSCD. E) Scatterplot showing a significant positive correlation between c+VEMP amplitudes and ABR amplitudes at 2 kHz (90 dB SPL). F) Scatterplot showing correlations between c+VEMP amplitudes (2 kHz, 90 dB SPL) and ABR amplitudes at 2 kHz (90 dB SPL) or 16 kHz (90 dB SPL). $*p \leq 0.05$, $**p \leq 0.001$, $***p \leq 0.0001$.

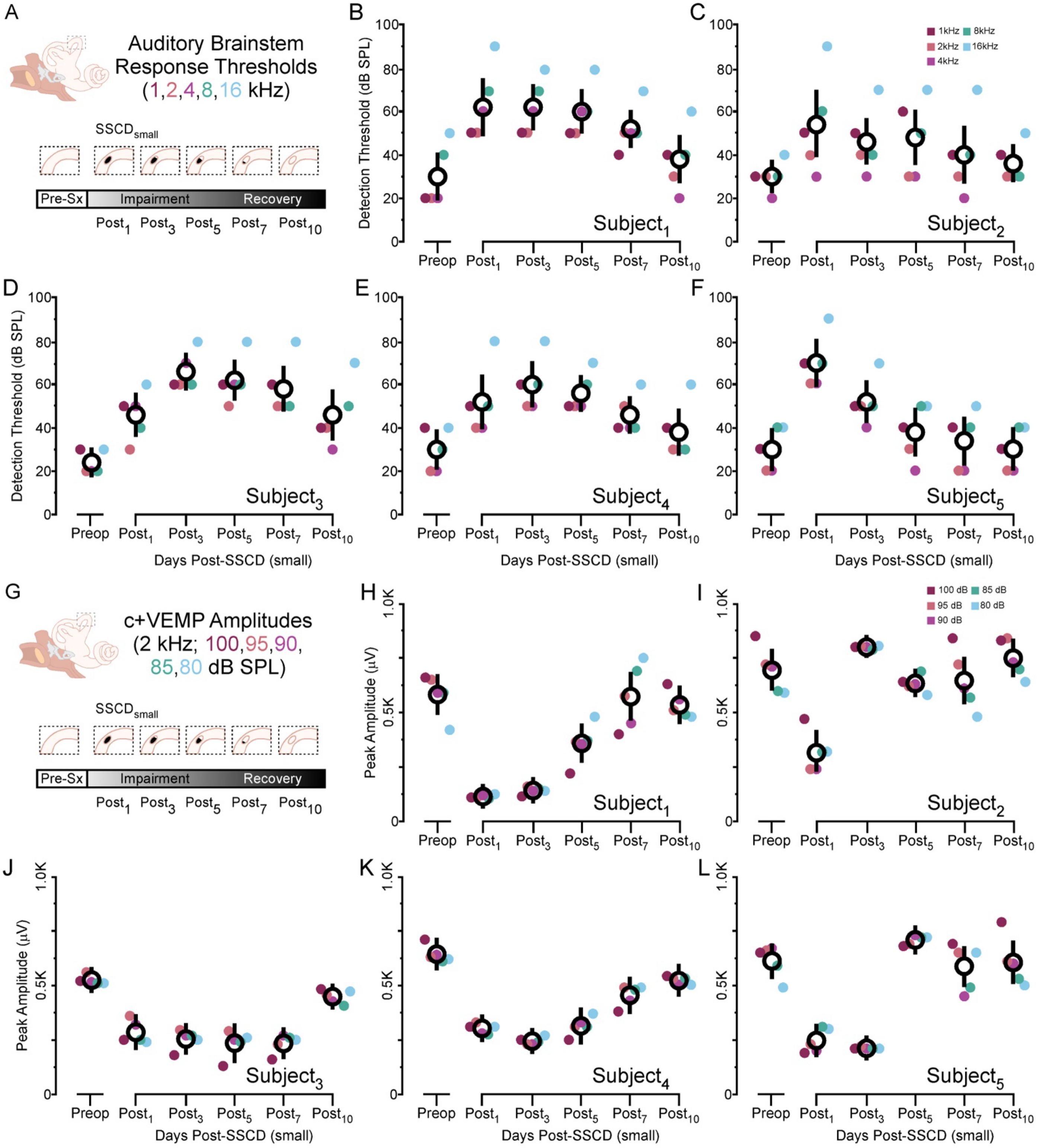

Supplementary Figure 3. Individual changes to ABR and c+VEMP thresholds after small SSCD over 10 days of recovery. A) Diagram showing the theoretical recovery of ABR thresholds over days (post-SSCD days 1, 3, 5, 7 and 10). B–F) Scatterplots showing the small SSCD-induced changes to ABR thresholds for days 1, 2, 4, 8, and 16 kHz on post-SSCD days 1, 3, 5, 7, and 10 for each animal. G) Diagram showing the theoretical recovery of c+VEMP amplitudes over days (post-SSCD days 1, 3, 5, 7, and 10). H–L) Scatterplots showing the small SSCD-induced changes to c+VEMP for 100, 95, 90, 85, and 80 dB SPL on post-SSCD days 1, 3, 5, 7, and 10 for each animal.

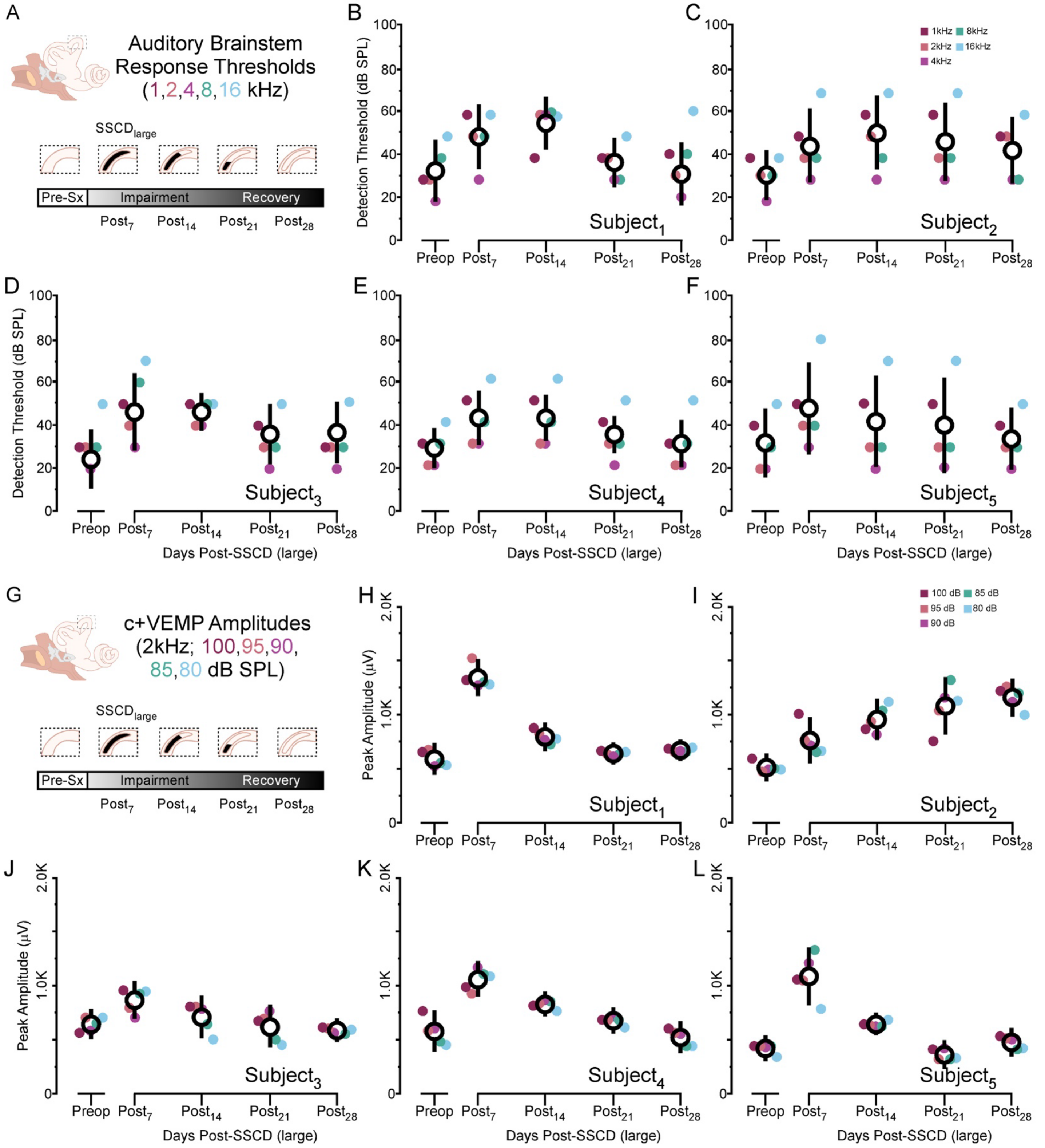

Supplementary Figure 4. Individual changes to ABR and c+VEMP thresholds after large SSCD over 28 days of recovery. A) Diagram showing the theoretical recovery of ABR thresholds over days (post-SSCD days 7, 14, 21, and 28). B–F) Scatterplots showing the large SSCD-induced changes to ABR thresholds for days 1, 2, 4, 8, and 16 kHz on post-SSCD days 7, 14, 21, and 28 for each animal. G) Diagram showing the theoretical recovery of c+VEMP amplitudes over days (post-SSCD days 7, 14, 21, and 28). H–L) Scatterplots showing the small SSCD-induced changes to c+VEMP amplitudes for 2 kHz at 100, 95, 90, 85, and 80 dB SPL on post-SSCD days 7, 14, 21, and 28 for each animal. $*p \leq 0.05$, $**p \leq 0.001$, $***p \leq 0.0001$.

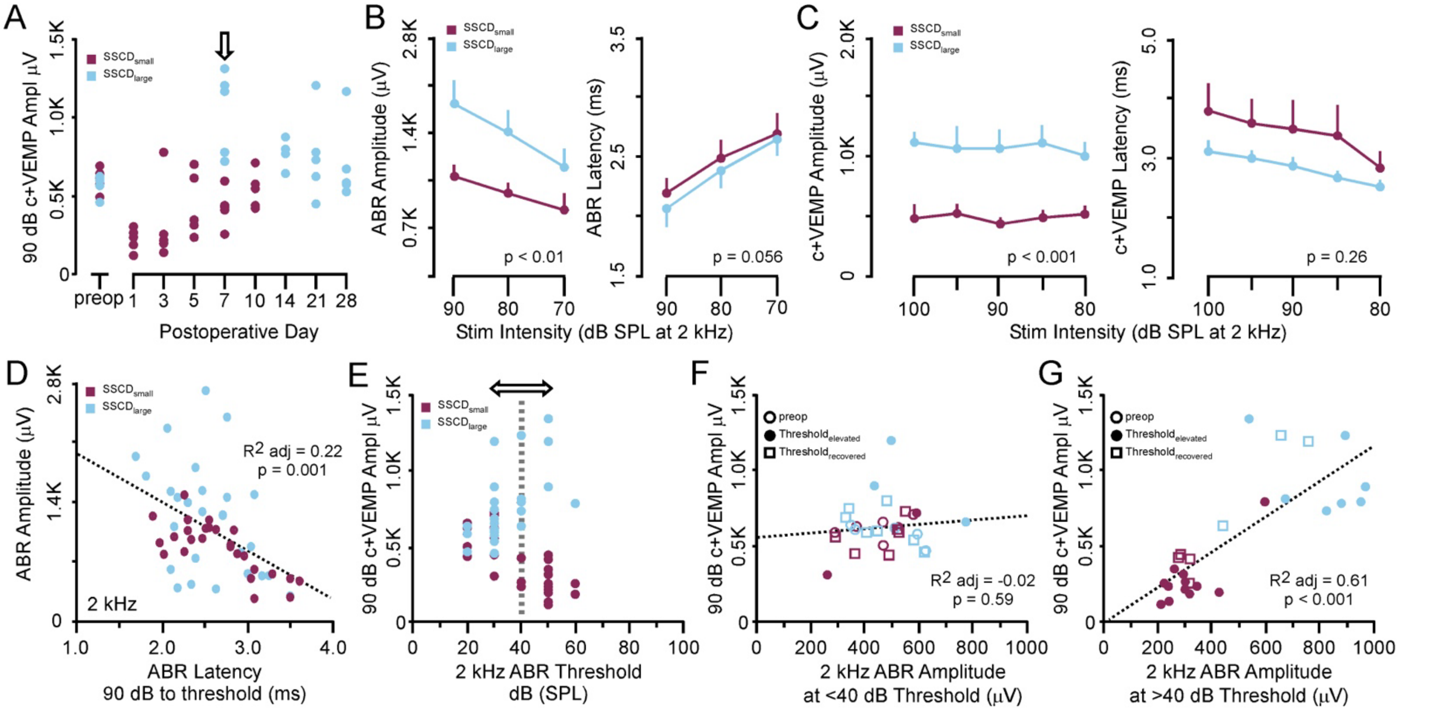

Supplementary Figure 5. Comparison of c+VEMP and ABR amplitudes based on SSCD size. A) Line chart showing the c+VEMP amplitudes for 2 kHz at 90 dB SPL for small and large fenestrations over all days of recovery. The arrow indicates the bimodal distribution seen on post-SSCD day 7 illustrating the polarized effect of small vs. large SSCD. B) Line plots comparing post-SSCD day 7 ABR amplitudes (left) and latencies (right) based on SSCD size. C) Line plots comparing post-SSCD day 7 c+VEMP amplitudes (left) and latencies (right) for 2 kHz at 100, 95, 90, 85, and 80 dB SPL based on SSCD size. D) Scatterplot comparing ABR amplitudes and latencies at 2 kHz across all days for small and large SSCD. E) Scatterplot showing c+VEMP amplitudes based on ABR thresholds at 2 kHz. The dashed line shows the threshold at which data becomes bimodally distributed. F) Scatterplot showing the correlation between c+VEMP amplitudes and ABR amplitudes at 2 kHz below the bimodal distribution threshold (< 40 dB SPL). G) Scatterplot showing the correlation between c+VEMP amplitudes and ABR amplitudes at 2 kHz above the bimodal distribution threshold (> 40 dB SPL).
